# Tomato and mini-cucumber tolerance to photoperiodic injury involves photorespiration and the engagement of nighttime cyclic electron flow from dynamic LEDs

**DOI:** 10.3389/fpls.2024.1384518

**Published:** 2024-05-22

**Authors:** Telesphore R. J. G. Marie, Evangelos Demos Leonardos, Naheed Rana, Bernard Grodzinski

**Affiliations:** Department of Plant Agriculture, University of Guelph, Guelph, ON, Canada

**Keywords:** photoperiodic injury, photorespiration, dynamic LEDs, cyclic electron flow, tomato, cucumber, continuous light, circadian rhythm

## Abstract

Controlled environment agriculture (CEA) is critical for achieving year-round food security in many regions of the world. CEA is a resource-intensive endeavor, with lighting consuming a large fraction of the energy. To lessen the burden on the grid and save costs, an extended photoperiod strategy can take advantage of off-peak time-of-day options from utility suppliers. However, extending the photoperiod limits crop production morphologically and physiologically if pushed too long. Here, we present a continuous-light dynamic light-emitting diode (LED) strategy (involving changes in spectra, intensity, and timing), that overcomes these limitations. We focused on tomato, a well described photoperiodic injury–sensitive species, and mini-cucumber, a photoperiodic injury-tolerant species to first assess morphological responses under control (16-h photoperiod, unchanging spectrum), constant (24-h photoperiod, unchanging spectrum), and two variations of a dynamic LED strategy, dynamic 1 (16-h “day”, 3-h “peak”, 8-h “night” spectra) and dynamic 2 (20-h “day”, 5-h “peak”, 4-h “night” spectra). Next, we tested the hypothesis of photorespiration’s involvement in photoperiodic injury by using a leaf gas exchange coupled with chlorophyll fluorescence protocol. We further explored Adenosine triphosphate (ATP): Nicotinamide adenine dinucleotide phosphate (NADPH) ratio supply/demand responses by probing photosynthetic electron flow and proton flow with the MultispeQ instrument. We found canopy architecture can be tuned by minor variations of the same dynamic LED strategy, and we highlight dynamic 1 as the optimal choice for both tomato and mini-cucumber as it improved biomass/architecture and first-yield, respectively. A central discovery was that dynamic 1 had a significantly higher level of photorespiration than control, for both species. Unexpectedly, photorespiration was comparable between species under the same treatments, except under constant. However, preliminary data on a fully tolerant tomato genotype grown under constant treatment upregulated photorespiration similar to mini-cucumber. These results suggest that photoperiodic injury tolerance involves a sustained higher level of photorespiration under extended photoperiods. Interestingly, diurnal MultispeQ measurements point to the importance of cyclic electron flow at subjective nighttime that may also partially explain why dynamic LED strategies mitigate photoperiodic injury. We propose an ontology of photoperiodic injury involving photorespiration, triose phosphate utilization, peroxisomal H_2_O_2_-catalase balance, and a circadian external coincidence model of sensitivity that initiates programmed cell death.

## 1 Introduction

Controlled environment agriculture (CEA), which includes indoor and greenhouse production systems, is becoming increasingly valuable for supplementing the nutritional needs of people across the world such as in northern regions with cold low-light winters, arid landscapes with drought-limiting field agriculture, tropical islands with high import expenses and hurricane susceptibility, and any metropolis with a dense urban population that creates food desert zones. However, CEA comes at a high energy cost. One of the largest consumers of energy in a CEA operation is lighting, with sole-source lighting in indoor facilities consuming much more than supplemental lighting in greenhouses that varies depending on geographical location and season (Harbick and Albright, 2016; Graamans et al., 2018; Weidner et al., 2021).

To tackle this obstacle, there have been recent advances in using an extended photoperiod strategy that takes advantage of off-peak time-of-day options provided by many utility suppliers to better manage the grid and costs (Tewolde et al., 2016; Hao et al., 2018). In fact, Ontario, Canada, can be one of the cheapest electricity sources in the world for large-scale operations if they follow the Industrial Conservation Initiative peak-shaving incentive (IESO, 2022; Ontario Energy Board, 2023; Hao, personal communications). Not only that, but the reason why peak costs are so high for utility providers is because the grid must be supplemented with fossil fuel generators during those times. During off-peak hours, the grid can be sustained by clean energy sources like hydro, wind, solar, and nuclear, which would, otherwise, be wasted if not used. Therefore, CEA would benefit, economically and environmentally, if it adheres to similar conservative energy-use policies.

Theoretically, if the supplemental light can be used for 24-h photoperiods, then the supplemental light intensity can be reduced by one-third while maintaining the same daily light integral (DLI) (Hao et al., 2018). However, a major limitation to the extended photoperiod strategy is the poor response that many species have to continuous light (e.g., eggplant, peanut, geranium, tomato, potato, lichen, and moss) (Velez-Ramirez et al., 2011). In the context of CEA-relevant species such as tomato, at worst, it causes photoperiodic injury, where yield is decreased and chlorotic leaves manifest (Garner and Allard, 1927; Dorais, 2003). At best, it is tolerated, as is the case for greenhouse cucumber (Hao et al., 2020; Lanoue et al., 2021). In many cases, it causes an overly compact plant architecture (Warner et al., 2023). For example, although continuous-light–tolerant tomato genotypes have been identified, continuous light decreases leaf area and height of these young transplants (Hao et al., 2018). Photoperiodic injury–tolerant tomato transplants must acclimate over 7 weeks by incrementally increasing the photoperiod from 16 h to 24 h, to effectively retain vegetative-generative balance (van Ieperen, 2016; Hao et al., 2018). Regardless of genotype, developmental stage, and species, the application of continuous light is not physiologically beneficial even though it is driving photosynthesis day and night.

If successful acclimation strategies can be identified for tomato (*Solanum lycopersicum* L. ‘Money Maker’), as a model photoperiodic injury–sensitive species and model tomato cultivar, then they would likely be useful for other species as well. Accordingly, we include a comparative study on mini-cucumber (*Cucumis sativus* L. ‘Beesan’) as a photoperiodic injury–tolerant species. We also report preliminary data on a completely photoperiodic injury–tolerant tomato genotype ‘UofGPIT.’

Both species were subjected to identical LED treatments that are modified versions of an alternating red-daytime dim-blue nighttime LED strategy that grew greenhouse tomato without injury (Lanoue et al., 2019). Photoperiodic injury in tomato is related to an arrhythmic circadian rhythm (Highkin and Hanson, 1954; Hillman, 1956; Velez-Ramirez et al., 2017a), and it is our perspective that efforts directed toward entraining the circadian rhythm will improve acclimation to extended photoperiod/continuous light (Marie et al., 2022). Furthermore, understanding circadian rhythm entrainment can help with guiding/compensating for the daily shifts in peak electrical pricing when growers would need daily fidelity in shifting supplemental lighting intensity without unbalancing the crop.

While constrained by the central motive of circadian entrainment, we can modify the alternating LED strategy to steer toward a better canopy architecture and measure the induced photosynthetic effects to gain insights that may further improve our understanding of photoperiodic injury tolerance. It would be helpful to identify specific photosynthetic traits that are diagnostic of successful acclimation to photoperiod extension. Or better yet, can we identify mechanisms that optimize photosynthesis under photoperiod extension and postulate future modifications that would engage them? Knowledge about these mechanisms would also aid in CEA-specific breeding efforts.

One proposition that can be largely agreed on is photoperiod extension imposing a state of excess energy. Depending on the source-sink balance and environmental factors, when there exists a state of excess incoming energy, different types of dissipative/protective mechanisms can be engaged in the short term. Long-term acclimation to excess excitation involves downregulation of source capacity and upregulation of sink capacity (Huner et al., 2003). The opposite response is induced under limited light availability. Collectively, these balancing responses are termed photostasis (Huner et al., 2003). In this context, we hypothesize photorespiration and its associated effects on metabolism/light reactions as a major mechanism involved with acclimating to extended photoperiods.

Photorespiration refers to a complex pathway that is initiated by oxygen (O_2_) competing against carbon dioxide (CO_2_) as a substrate with ribulose bisphosphate (RuBP) catalyzed by RuBP carboxylase/oxygenase (RuBisCO), creating an alternative pathway at the first step of the Calvin cycle (Ogren, 1984). Oxygenation of RuBP results in the production of phosphoglycolate and one phosphoglycerate, instead of two phosphoglycerates from RuBP carboxylation. In C3 plants, phosphoglycolate is eventually converted into phosphoglycerate to contribute to the Calvin cycle after several steps progressing through the chloroplast, peroxisomes, mitochondria, and back. A detailed description of these steps is not the focus of this manuscript, but some of them are highlighted as having significant implications for photoperiod extension.

Photorespiration has been reported to be an important energetic sink mechanism being used under drought stress (Valentini et al., 1995; Guan and Gu, 2009), salt stress (Hannachi et al., 2022), and combined high temperature/light stress (Osei-Bonsu et al., 2021). However, Smith et al. (2023) found that photorespiration is not a short-term energy dissipative pathway that directly alleviates photosystem II (PSII) damage. Rather, photorespiration has a role in sustaining the Calvin cycle that allows for the timely synthesis of D1 protein slotted for PSII repair (Takahashi et al., 2007). It sustains the Calvin cycle by ensuring sufficient inorganic phosphate (P_i_) substrate for ATP turnover. The relationship between photorespiration and Calvin cycle turnover can be best observed as triose phosphate utilization (TPU) limitation (Sharkey, 1985; McClain and Sharkey, 2019). Photorespiratory-mediated P_i_ release, which has a positive effect under TPU-limited conditions, also has an impact on ATP: NADPH stoichiometry. It has been established that increased relative levels of photorespiration increases relative ATP demand, changing the ATP: NADPH demand stoichiometry, which needs to be balanced by increasing the ATP: NADPH ratio supply via upregulating ATP-generating (cyclic) or NADPH-consuming/alternative electron sink (pseudo-cyclic) mechanisms (Kramer and Evans, 2011).

In addition to a hypothesized increase in photorespiration under successful acclimation to extended photoperiods, we also hypothesize an associated increase in cyclic/pseudo-cyclic mechanism to sustain it. Our objectives were to 1) measure basic morphological and biomass partitioning in tomato and mini-cucumber under dynamic LEDs, 2) employ a combined gas exchange and fluorescence protocol to quantify photorespiration, 3) probe ATP balancing mechanisms during the short-term diurnal phases of the dynamic LED treatments and the long-term acclimated steady state, and 4) assess the similarities and differences between photoperiodic injury–sensitive tomato ‘Money Maker’ and photoperiodic injury–tolerant mini-cucumber ‘Beesan’ (along with supplemental comparisons to a tolerant tomato genotype).

## 2 Methods

### 2.1 Plant material and growth conditions

At the University of Guelph, Ontario, Canada, tomato ‘Money Maker’ was sown and placed in a growth chamber (Conviron, Winnipeg, Canada) for 2 weeks, under humidity domes with environmental conditions set to 25°C (day/night) and fluorescent lighting (5,000-K white, single pin T12 tubes, Sylvania Inc., Wilmington, MA, USA) set to 150 µmol m^−2^ s^−1^ photosynthetic photon flux density (PPFD) for 16-h light/8-h dark photoperiod. Mini-cucumber ‘Beesan’ was sown under identical conditions but for 1 week. The most vigorous plants were then transplanted into 15-cm-wide plastic square pots filled with standard potting mix (Sungro professional growing mix #1, Soba Beach, AB, Canada) and transferred into a “nursery” growth chamber (GC-20 Bigfoot series, Biochamber, Winnipeg, Canada) equipped with LEDs (see below lighting treatment) for 7 days set to 21°C (day/night), 65% relative humidity, 300 µmol m^−2^ s^−1^ PPFD, and 16-h light/8-h dark photoperiod. Afterward, plants were transferred to treatment chambers (GC-20 Bigfoot series, Biochamber, Winnipeg, Canada), all under identical environmental conditions (previously calibrated with external sensors) except for the lighting treatments described below. Fertigation was supplied as needed with 20–8-20 fertilizer (Plant Products Inc., Leamington, ON, Canada) mixed in regular tap water (Guelph, Ontario tap water is relatively high in carbonates, pH approximately 7, electrical conductivity (EC) approximately 0.85 mS/cm) and adjusted to a pH of 5.6 with phosphoric acid to a final EC of 1.75 mS/cm. Leaf gas exchange and fluorescence measurements were done 42 days after sowing (DAS) for tomato and 35 DAS for mini-cucumber, targeting the third true leaf. After photosynthetic measurements, an additional 4 days were given until destructive analysis (all on the same day of the given week). At this relatively large transplant age, ‘Money Maker’ only had small primordial floral development, but mini-cucumber ‘Beesan’ had several fruits of harvestable size. To assess this early yield, no thinning was performed prior to destructive harvest and only fruits that were >5-cm long were included in the weight (in some cases, there were a dozen or more<2-cm fruits that were not included in weight measurements).

### 2.2 Lighting treatments

**Figure 1:**
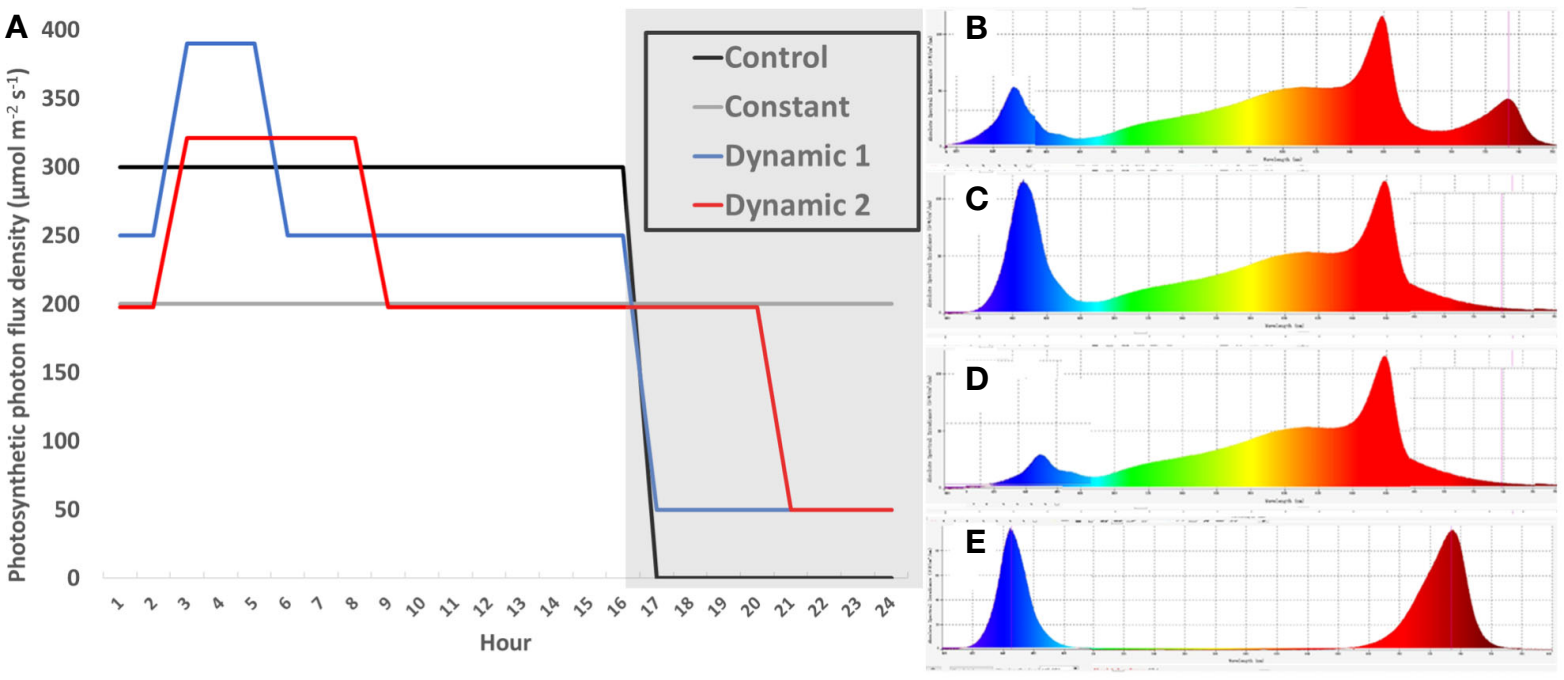
Schedule of light treatments. A single diurnal cycle is represented by non-shaded (day) and shaded bars (night) (hour 1 = 8 am) **(A)**. Light intensity is plotted across time-of-day for each light treatment, which are sum to equivalent DLIs (17.28 mol m^−2^ d^−1^) **(A)**. Control (solid line) and constant (double thin line) both had the same relative spectra for their entire photoperiod **(B)**, consisting of cool-white supplemented with red and far-red spectra (CW + R + FR). Dynamic 1 and dynamic 2 had a “day” spectrum of warm-white plus red (WW + R) for the first 2 h after subjective dawn **(D)**. They then received a “peak” spectrum **(C)** that supplemented 140 PPFD of pure blue on top of day spectrum (WW + R + Bl) for 3 h and 5 h, respectively. After the peak phase was finished, they returned to “day” spectrum until the end of their 16-h and 20-h photoperiods, respectively. During subjective night, they each received dim-blue and far-red (Bl + FR) **(E)**, although the intensity of far-red was higher in dynamic 2 to ensure all treatments received the same dose of FR (**Supplementary Material 1**).

Biochambers had four independently controllable light banks that were each equipped with T5-type ballast compatible replacement LEDs tubes (red LEDs, SKU F54T5HO-LED36R, Growlights Canada Inc., Beamsville, ON, CAN; blue LEDs, SKU F54T5HO-LED36B, Growlights Canada Inc., Beamsville, ON, CAN; 3,500-K white, LED25WT5HO/46/835-G8DR, Lumenco Inc., Trois-Rivières, QC, CAN; and 5,000-K white, LED25WT5HO/46/850-G8DR, Lumenco Inc., Trois-Rivières, QC, CAN). Depending on the treatment, different designated light banks were use to supply the needed spectrum that had either control (steady unchanging spectrum for 16-h photoperiod), constant (steady unchanging spectrum for 24-h photoperiod), dynamic 1 (changing spectrum and intensity depending on time of day), or dynamic 2 (changing spectrum and intensity depending on time of day) (**Figure 1**). The spectrum was changed by using biochamber control software for timing different light banks with the addition of separate far-red LED fixtures on a timer (FGI Far Red, FARREDLB, Forever Green Indoors Inc., Seattle, WA, USA). Total DLI and far-red DLI were the same across all treatments; however, blue DLI was only the same between control and constant or dynamic 1 and dynamic 2 (**Supplementary Material 1**).

### 2.3 Leaf gas exchange and chlorophyll fluorescence

Simultaneous gas exchange and chlorophyll fluorescence were performed using two LiCor 6400 (LI-COR Biosciences, NE, USA) instruments side by side, each with the 6400–40 leaf chamber fluorometer head. All measurements were done with the LiCor 6400 heads fixed inside a growth chamber to maintain ambient lighting and temperature around the whole plant while protocols were being done on the individual leaf.

A quick transition between 21% and 2% O_2_ (**Supplementary Material 2**) was done to follow the photorespiration protocol explained by Bellasio et al. (2014). The protocol provides the necessary variables to derive RuBisCO activities (**Equation 1**). Photorespiration is calculated as the ratio between RuBisCO oxygenase activities (V_O_) and RuBisCO carboxylation activities (V_C_), V_O_/V_C_. The equation requires gross assimilation (GA under 21% O_2_ and GA under low O_2_) as inputs, and, in our case, we used dark respiration (rather than day respiration estimation techniques) to calculate it from net assimilation (and net assimilation under low O_2_) as it was simply more convenient in our protocol. In support of this decision, variations in day respiration estimates only sway the results by approximately 4% according to the sensitivity analysis done by Bellasio et al. (2014). The equation also requires PSII photochemical quantum yield (YII) under ambient O_2_ and low O_2_.


(1) $$\frac{V_{O}}{V_{C}}=\frac{2{\text{GA}}_{\text{Low O}2}\frac{Y\left(II\right)}{Y{\left(II\right)}_{Low\;O2}}-2\text{GA}}{{\text{GA}}_{\text{Low O}2}\frac{Y\left(II\right)}{Y{\left(II\right)}_{Low\;O2}}+2\text{GA}}$$

### 2.4 MultispeQ measurements

The MultispeQ (PhotosynQ Inc., MI, USA) is a leaf spectrophotometer/fluorometer designed for open-source research (Kuhlgert et al., 2016). The programmability and customizations available make it a very useful tool. The “Photosynthesis RIDES 2.0” protocol was selected as it has very high throughput and provides over 14 photosynthetic response variables in approximately 1 min. An overview of the equations used in the protocol can be found on PhotosynQ’s webpage “documentation” under the subsection “references and parameters” (Kramer et al., 2023). The protocol and associated macro were used in their original form, without modifications. The protocol also offers the measurement of broadband electrochromic shift (ECS) under dark-interval relaxation kinetic (DIRK) assays that can be used in combination with fluorescence techniques, described by Baker et al. (2007). Together, using the “Photosynthesis RIDES 2.0” protocol, it is possible to probe both electron flow and proton flow under steady-state light-adapted conditions. Two parameters were calculated separately in excel that combine fluorescence and absorption parameters provided by the MultispeQ according to Baker et al. (2007). Proton motive force from linear electron flow (LEF) only (pmf_LEF_) was calculated by dividing the fluorescence-based parameter LEF by the ECS DIRK absorption–based parameter that estimates ATP synthase conductivity/activity (gH^+^). Proton pumping by cyclic electron flow (CEF) (ν_H+_LEF^−1^) was calculated by dividing the ECS DIRK absorption–based relative proton flux (ν_H+_) by fluorescence-based LEF and multiplying it by 1,000 (Avenson et al., 2005; Baker et al., 2007). Apparent conductance of cytochrome b_6_f was calculated by dividing LEF by the portion of closed PSII reaction centers, where open reaction centers follow the lake model (qL), giving a parameter notation [LEF (1 − qL)^−1^] (Johnson et al., 2021).

Depending on the experimental design, either the plants were transferred to a growth chamber for measurements under identical environmental conditions (PPFD, RH%, and temperature) (for 3-week acclimated representative steady state) or the measurements were taken under the actual conditions for the designated treatments (with different PPFD/spectrum) (for short-term time-course experiment).

Note that, throughout the time course, the plants acclimated to changes in light intensity and quality, but our measurements were done under a common light quality (red, 660 nm) that only responds to ambient light intensity sensed on the top of the MultispeQ. This is simply the programming of the Photosynthesis RIDES 2.0 protocol. Future studies could customize light quality differences, but these results serve as a good indication of photosynthetic mechanisms using an unaltered protocol that is widely available and repeatable.

### 2.5 Whole-plant biomass and partitioning

Plants were harvested destructively for total above-ground biomass, biomass partitioning (between leaves, petioles, and stems), and plant architecture (stem height, leaf surface area, specific leaf area, etc.). Partitioned plant materials were dried in an oven to get the measurement of dried weights. Leaf area was measured by using a personal smartphone, with the Easy Leaf Area app (Easlon and Bloom, 2014), rigged to a retort stand to maintain consistent lighting and distance from a black cloth background with a 4-cm^2^ red cardboard square for automatic scaling in the app. Once the best green/blue/red scales were adjusted to ensure uniform leaf and red square highlighting, the same settings were used for all future pictures through the app.

### 2.6 Statistics

Statistical analyses were done using Proc Glimmix in SAS Studio 3.81. One-way analysis of variance (ANOVA) was performed on the destructive whole-plant datasets according to a completely randomized block design, with light treatment as fixed factor and random factor blocked by week of sowing. Tomato had 10–12 samples over 5 successive weeks of sowing. Week of sowing was blocked as it contained a high amount of variability due to slight age differences between cohorts of sowing and other unknown random factors. When ANOVAs were significant (p< 0.05), means comparisons were performed using Tukey–Kramer adjustment, testing the significant difference (p< 0.05).

## 3 Results

### 3.1 Tomato canopy architecture, biomass, and partitioning

Over a 3-week course of treatment, constant light accumulated the least biomass resulting in a significant difference in total biomass compared to all other treatments (**Table 1**). Specific leaf area was the highest under constant light compared to all other light treatments, and, inversely, the leaf mass per area was significantly lower under constant light. Biomass partitioning analysis found that constant light allocated more dry weight to stem mass fraction at the expense of leaf mass fraction, both significantly different from control. Leaf area under constant light was not significantly different from control or dynamic 2, but it was significantly lower than dynamic 1. The same was true for height.

**Table 1:** Biomass and partitioning traits measured from destructive analysis comparing tomato ‘Money Maker’ grown under different photoperiod extension strategies in growth chambers.

Light treatment		Control	Constant	Dynamic 1	Dynamic 2
*Total Biomass*	*(g)*	10.63 ± 1.16 B	7.86 ± 1.16 C	12.90 ± 1.14 A	11.73 ± 1.14 AB
*Height*	*(cm)*	28.0 ± 2.1 B	30.3 ± 2.1 B	40.1 ± 2.0 A	29.9 ± 2.0 B
*Leaf area*	*(m^2^)*	0.206 ± 0.018 AB	0.183 ± 0.018 B	0.230 ± 0.018 A	0.186 ± 0.018 B
*Specific leaf area (m^2^ kg^−1^)*		29.0 ± 1.9 B	37.6 ± 1.9 A	27.5 ± 1.8 B	24.0 ± 1.8 B
*Leaf mass per area*	*(g m^−2^)*	35.6 ± 1.9 B	27.7 ± 1.9 C	37.1 ± 1.8 B	43.0 ± 1.8 A
*Stem mass fraction*	*(g g^−1^)*	0.16 ± 0.007 C	0.19 ± 0.007 AB	0.20 ± 0.007 A	0.17 ± 0.007 BC
*Petiole mass fraction*	*(g g^−1^)*	0.16 ± 0.003 A	0.17 ± 0.003 A	0.14 ± 0.003 B	0.15 ± 0.003 B
*Leaf mass fraction*	*(g g^−1^)*	0.67 ± 0.008 AB	0.64 ± 0.008 C	0.66 ± 0.008 BC	0.68 ± 0.008 A

Mass fractions (in dry weight) were calculated by dividing the organ of interest by total above-ground biomass. Means and standard error (n = 10–12) are presented with letters to denote if a significant difference was found using least square means with Tukey–Kramer adjustment (p< 0.05), same letters are not significantly different from each other.

**Figure 2:**
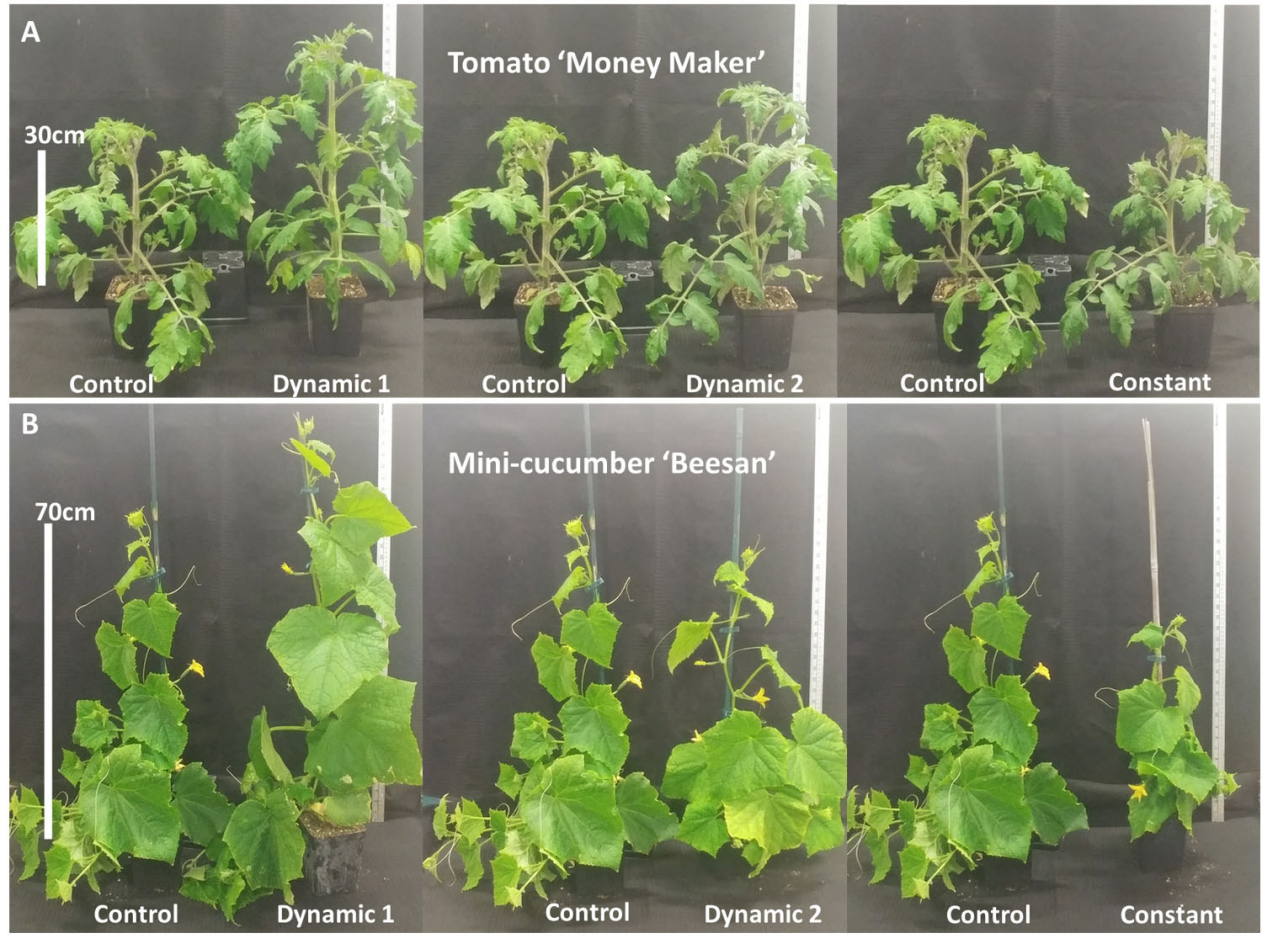
Representative size and architecture of **(A)** tomato ‘Money Maker’ and **(B)** mini-cucumber ‘Beesan’ after being exposed to indicated light treatments for 3 weeks. Both species are represented by spliced images, with the same control plant that was not moved from its position in each cropped section.

Dynamic 1 had a significantly higher total biomass than control and constant but not significantly different from dynamic 2. Dynamic 1 was the tallest of all light treatments (**Figure 2**). Biomass partitioning trends for dynamic 1 showed a higher stem mass fraction than control at the expense of petiole mass fraction, with no significant difference in leaf mass fraction. Specific leaf area and leaf mass per area were not significantly different from control; however, they were both different from constant.

Dynamic 2 accumulated more total biomass than constant but was not significantly different from control or dynamic 1. Leaf mass per area was higher in dynamic 2 compared to all other treatments, but specific leaf area did not reflect this difference. Dynamic 2 height, leaf area, and stem mass fraction were not significantly different from control or constant. Dynamic 2 petiole mass fraction was lower than control and constant but the same as dynamic 1. Leaf mass fraction was higher than constant and dynamic 1 but not significantly different from control.

On a qualitative visual level of leaves, dynamic 1 did not have any observable chlorosis (nor did control), whereas constant had a severe chlorosis that was variable in degree across replicates. Dynamic 2 had a very minor form of chlorosis that was almost imperceptible, and it was not noticed in some replicates.

### 3.2 Mini-cucumber canopy architecture, biomass, and partitioning

Overall, dynamic 1 had higher total biomass and greater height than constant and dynamic 2 treatments, but neither variable was significantly different from control (**Table 2**). Interestingly, leaf morphology looked different between dynamic 1 and control; however, it was not quantified (**Figure 2**).

**Table 2:** Biomass and partitioning traits measured from destructive analysis comparing mini-cucumber ‘Beesan’ grown under different photoperiod extension strategies in growth chambers.

Light treatment		Control	Constant	Dynamic 1	Dynamic 2
*Total biomass*	*(g)*	17.05 ± 1.09 AB	11.05 ± 1.09 C	18.17 ± 1.09 A	15.43 ± 1.07 B
*Yield*	*(g)*	70.42 ± 13.36 B	31.17 ± 13.36 C	93.77 ± 13.35 A	52.42 ± 13.20 BC
*Height*	*(cm)*	81.09 ± 4.12 A	54.72 ± 4.12 B	70.44 ± 4.12 A	52.08 ± 3.98 B
*Leaf area*	*(m^2^)*	0.290 ± 0.018 A	0.225 ± 0.018 B	0.283 ± 0.018 A	0.228 ± 0.017 B
*Specific leaf area (m^2^ kg^−1^)*		30.22 ± 1.40 A	32.59 ± 1.40 A	31.58 ± 1.40 A	23.88 ± 1.32 B
*Leaf mass per area*	*(g m^−2^)*	33.90 ± 1.47 B	31.06 ± 1.47 B	31.98 ± 1.47 B	42.07 ± 1.1.40 A
*Stem mass fraction*	*(g g^−1^)*	0.18 ± 0.01 A	0.18 ± 0.01 A	0.17 ± 0.01 A	0.14 ± 0.01 B
*Petiole mass fraction*	*(g g^−1^)*	0.058 ± 0.003 C	0.054 ± 0.003 C	0.078 ± 0.003 A	0.068 ± 0.003 B
*Leaf mass fraction*	*(g g^−1^)*	0.57 ± 0.03 B	0.64 ± 0.03 A	0.50 ± 0.03 C	0.62 ± 0.03 AB
*Harvest index*	*(g g^−1^)*	0.19 ± 0.03 AB*	0.12 ± 0.03 C	0.25 ± 0.03 A*	0.17 ± 0.03 BC

Mass fractions (in dry weight) were calculated by dividing the organ of interest by total above-ground biomass. Yield was the first harvest from unpruned 6- to 7-week-old plants (fresh weight). Means and standard error (n = 8–9) are presented with letters to denote if a significant difference was found using least square means with Tukey–Kramer adjustment (p< 0.05), same letters are not significantly different from each other.

*Harvest index difference between control and dynamic 1 (p = 0.0921).

**Figure 3:**
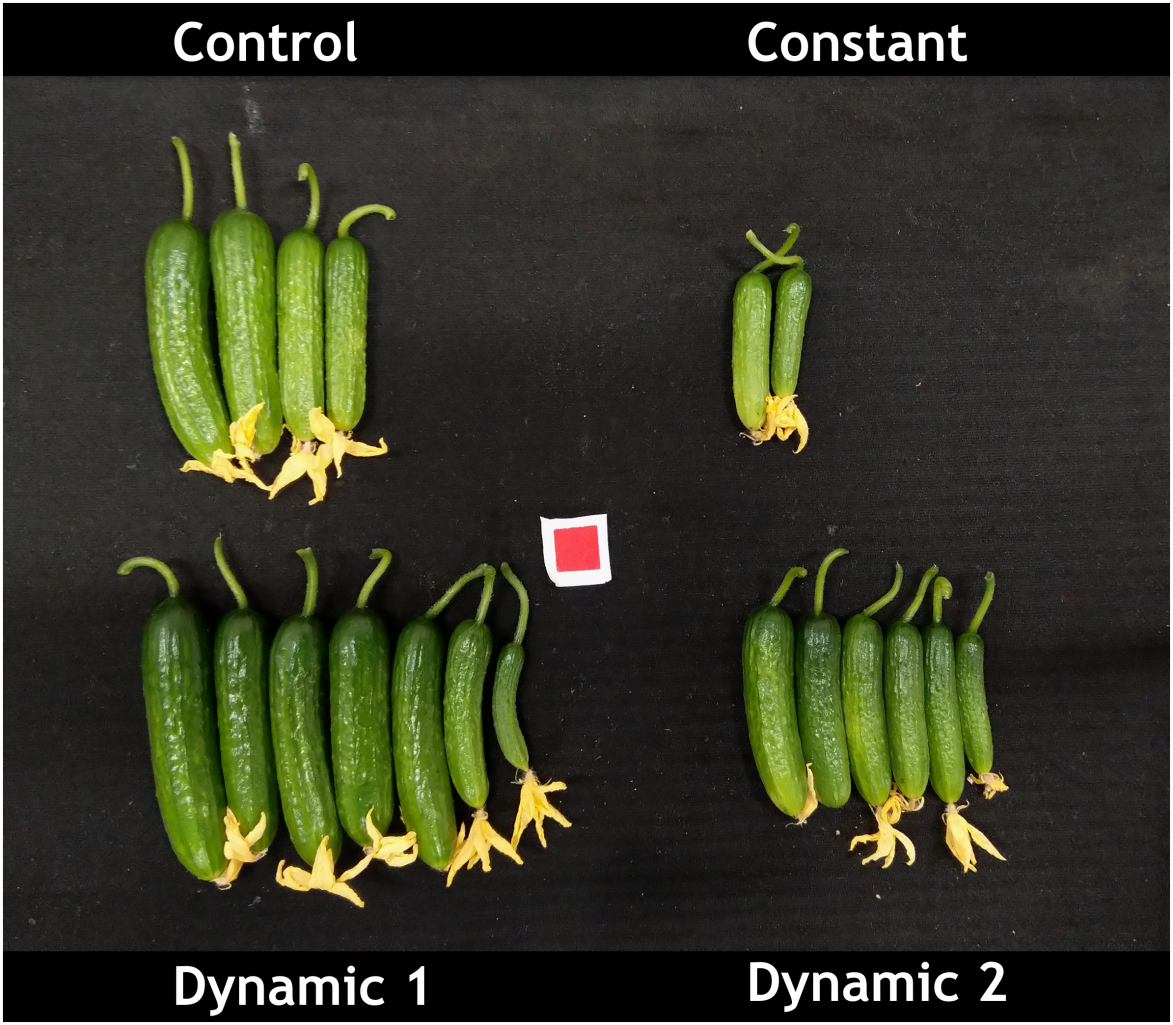
Representative image of mini-cucumber ‘Beesan’ first harvest (from two plants) after approximately 3 weeks of growth under the indicated light treatments. Dynamic 1 had the highest yield as depicted in the image, and it was statistically significant. Note that both dynamic LED treatments induced a greener fruit, which was not quantified in the present study.

Dynamic 1 had the highest yield compared to all other treatments. Furthermore, the fruit in both dynamic treatments looked much greener and of higher quality than control and constant (**Figure 3**). Biomass partitioning showed that dynamic 1 allocated more dry matter to petiole mass fraction than any other treatment and allocated less to leaf mass fraction than the others. Dynamic 1 had the highest harvest index; however, it was not significantly different from control unless the alpha value is relaxed to 0.10 (p = 0.0921), which would help interpretation considering the higher yield and significantly less allocation to leaf mass fraction while maintaining similar total biomass.

Constant-light treatment had the lowest total biomass compared to all other treatments. Yield was significantly less under constant light than the other treatments, except for dynamic 2. Height and leaf area showed the same trend, with constant light being less than control and dynamic 1, but not significantly different from dynamic 2. Biomass partitioning shows constant light induced more dry matter to be allocated to leaf mass fraction than harvest index compared to control.

Dynamic 2 had a total biomass that was significantly higher than constant. However, height and leaf area were both comparable to constant. The extra biomass was observed to come from an increase in leaf mass per area, which was significantly higher than all other treatments. Dynamic 2 diverted the most partitioning away from stem mass fraction compared to all treatments. Interestingly, it had an intermediate level of partitioning to petiole mass fraction that was significantly greater than control, but significantly less than dynamic 1. It also retained more partitioning to leaf mass fraction than dynamic 1, being comparable to both constant and control. Finally, dynamic 2 yield and harvest index were significantly lower than dynamic 1 and not significantly different from either control or constant.

### 3.3 Photosynthesis and photorespiration of tomato and mini-cucumber under ambient conditions after 3-week acclimation

The initial survey measurements described in Methods section “Leaf gas exchange and chlorophyll fluorescence” were designed to follow a high throughput screening method for determining rates of photorespiration (Bellasio et al., 2014). The intention was to quantify photorespiration at ambient conditions for each lighting treatment.

**Figure 4:**
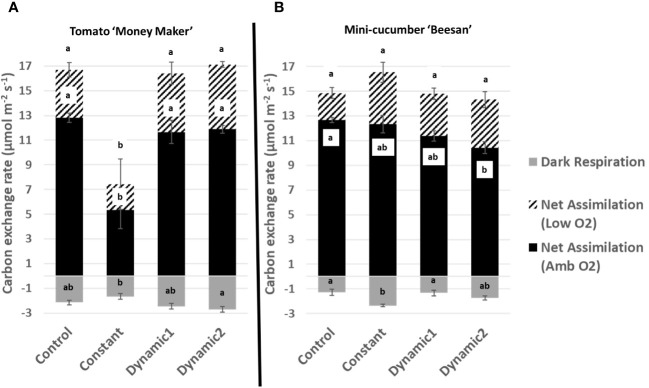
Carbon exchange rates measured under ambient conditions from tomato ‘Money Maker’ **(A)** and mini-cucumber ‘Beesan’ **(B)** leaves acclimated to different lighting treatments. For tomato, all treatments, except constant, are not significantly different from each other. For mini-cucumber, constant seems to have a higher net assimilation (low O_2_), but it is not significant. However, constant has a larger dark respiration than all other treatments. Dynamic 2 has a lower net assimilation (ambient O_2_) than control, but it is not significantly different from constant and dynamic 1. Means and standard error (n = 4) are presented with letters to denote if a significant difference was found using least square means with Tukey–Kramer adjustment (p< 0.05), same letters mean they are not significantly different from each other.

Upon a standard gas exchange analysis of dark respiration, net assimilation (ambient O_2_), and net assimilation (low O_2_), the only apparent significant difference across treatments is the severe decline found under constant light for tomato (**Figure 4**). Constant-light treatment for cucumber, however, maintained all parameters similar to control with the exception of having significantly greater respiration in the dark. Although net assimilation under low O_2_ seems higher under constant for cucumber, the variability between samples masks any significant differences. Net assimilation under ambient O_2_ was significantly lower under dynamic 2 than control for cucumber but was not different under low O_2_.

**Figure 5:**
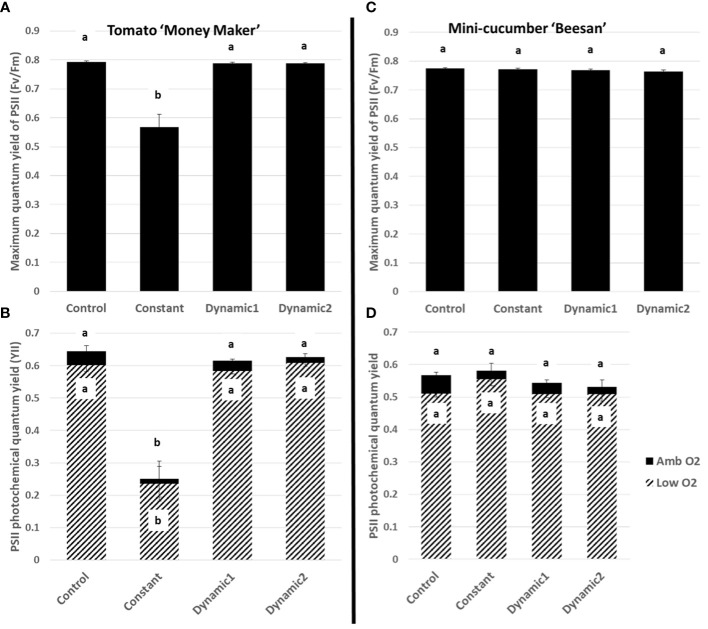
Fluorescence parameters measured under ambient conditions from tomato leaves acclimated to different lighting treatments. Maximum quantum yield of PSII (Fv/Fm) **(A, C)** and PSII photochemical quantum yield (YII) **(B, D)**. For tomato, all treatments, except constant, are not significantly different from each other. For cucumber, constant retained PSII function similar to all other light treatments. Means and standard error (n = 4) are presented with letters to denote if a significant difference was found using least square means with Tukey–Kramer adjustment (p< 0.05), same letters are not significantly different from each other.

The same differences across treatments, relative to constant-light treatment depending on species, were found from a standard chlorophyll fluorescence analysis of maximum quantum yield (Fv/Fm) of PSII and PSII photochemical quantum yield (YII), where only constant-light treatment for tomato was significantly lower (**Figure 5**). These results on their own are commonly reported in the literature and used to assess the effectiveness of treatments.

**Figure 6:**
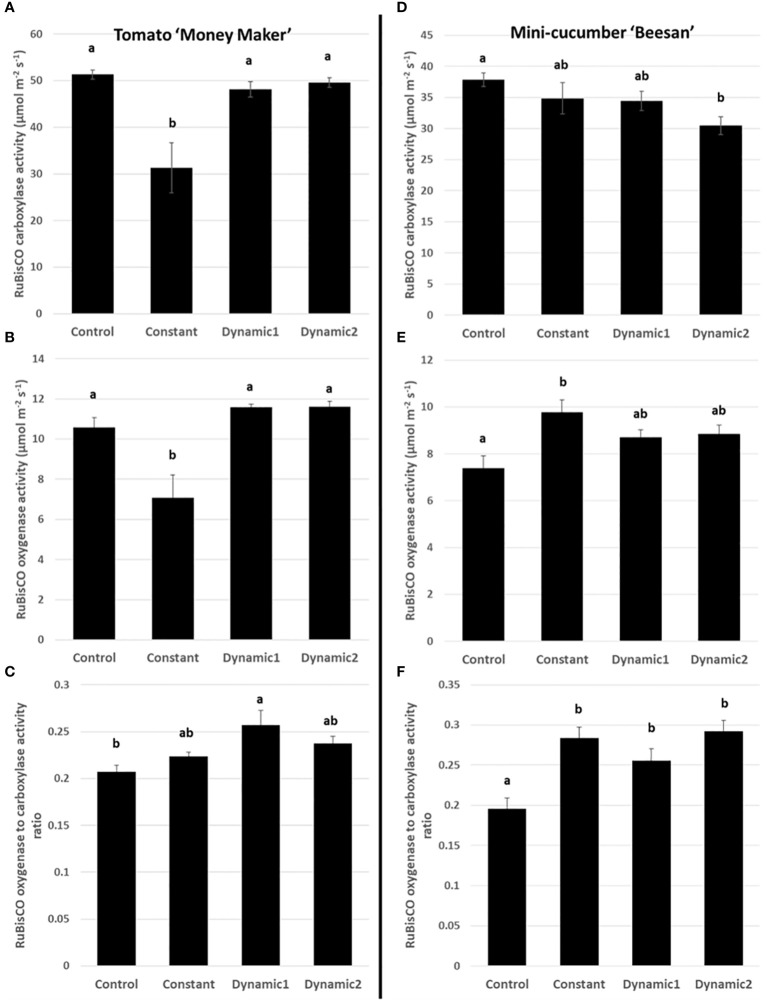
RuBisCO carboxylase **(A, D)** and oxygenase **(B, E)** activities under ambient conditions (V_C_ and V_O_) and photorespiration estimated by their ratio (V_O_/V_C_) **(C, F)**. Constant-light treatment for tomato had a significantly lower V_C_ and V_O_ than control. Dynamic 1 and dynamic 2 were similar to control in V_C_ and V_O_, and both were significantly higher than constant. V_O_/V_C_ was significant different between control and dynamic 1. Cucumber, however, maintains higher V_O_/V_C_ under constant (and both dynamic LED treatments) than control. Means and standard error (n = 4) are presented with letters to denote if a significant difference was found using least square means with Tukey–Kramer adjustment (p< 0.05), same letters are not significantly different from each other.

All treatments, except constant light, had comparable RuBisCO carboxylation activities (V_C_) and RuBisCO oxygenase activities (V_O_) for tomato (**Figure 6**), whereas cucumber V_C_ was significantly lower in dynamic 2 than control and V_O_ was significantly higher in constant than control. For tomato, the RuBisCO oxygenase to carboxylation activity ratio (V_O_/V_C_) shows a significant increase in dynamic 1 compared to control, whereas V_O_/V_C_ in constant-light treatment balanced out to be equivalent to the other treatments, which makes sense considering it was equally depressed in both V_C_ and V_O_. For cucumber, V_O_/V_C_ was significantly higher in all photoperiod extension treatments than control.

### 3.4 Long-term MultispeQ-derived photosynthetic variables of tomato

After 3 weeks of acclimation to respective lighting treatments, tomato plants were transferred (at approximately 2 pm) to a common growth chamber so that they all may be measured under similar conditions. In terms of fluorescence-based parameters provided by the MultispeQ instrument (**Table 3**), all analyzed light treatments had comparable LEF. PSII maximum efficiency (Fv’/Fm’) and non-photochemical quenching (NPQt) were significantly lower and higher, respectively, in dynamic 2 compared to control and dynamic 1. Although NPQt was reported, the protocol does not distinguish between quenching mechanisms possible through time-dependent quenching assays for q_E_, q_T_, and q_I_. Dynamic 1 had a significantly lower fraction of open PSII reaction centers (qL) than control, but dynamic 2 was not significantly different from either. Dynamic 1 also had a significantly lower apparent conductance of cytochrome b_6_f (Cyt b_6_f) [LEF (1 − qL)^−1^], a parameter derived from Johnson et al. (2021).

**Table 3:** A comparison of photosynthetic variables from tomato leaves grown under different photoperiod extension strategies in growth chambers acquired with MultispeQ using the protocol “Photosynthesis RIDES 2.0”.

Light treatment		Control	Dynamic 1	Dynamic 2
*Linear electron flow*	*LEF*	64.38 ± 1.192 A	61.40 ± 0.766 A	61.64 ± 0.801 A
*PSII maximum efficiency*	*Fv’/Fm’*	0.783 ± 0.002 A	0.781 ± 0.001 A	**0.772 ± 0.002 B**
*Non-photochemical quenching*	*NPQt*	0.352 ± 0.018 B	0.366 ± 0.010 B	**0.443 ± 0.020 A**
*Fraction of PSII centers in open state*	*qL*	0.623 ± 0.015 A	**0.551 ± 0.010 B**	0.569 ± 0.018 AB
*Apparent conductance of Cyt b_6_f to linear electron flow*	*LEF (1 − qL)^−1^*	173.6 ± 10.70 A	**138.2 ± 4.069 B**	146.9 ± 8.121 AB
*Quantum yield of PSII (fraction of excitons driving LEF)*	*ϕ_PSII_*	0.691 ± 0.006 A	**0.662 ± 0.004 B**	**0.657 ± 0.008 B**
*Fraction of excitons dissipated through regulated non-photochemical quenching*	*ϕ_NPQ_*	0.080 ± 0.004 B	0.090 ± 0.002 B	**0.105 ± 0.005 A**
*Fraction of excitons dissipated through non-regulated mechanisms*	*ϕ_NO_*	0.228 ± 0.004 B	**0.247 ± 0.003 A**	0.238 ± 0.005 AB
*Steady-state relative thylakoid proton efflux (ATP synthase conductivity/activity)*	*g_H+_*	143.9 ± 9.789 B	**186.9 ± 8.300 A**	133.8 ± 8.272 B
*Relative proton flux (H^+^/ATP ratio multiplied by ATP synthesis rate)*	*ν_H+_*	0.084 ± 0.003 A	0.082 ± 0.002 A	0.076 ± 0.003 A
*Proton pumping by cyclic electron flow (×1,000)*	*ν_H+_ LEF*^−^*^1^*	1.300 ± 0.045 A	1.344 ± 0.049 A	1.241 ± 0.054 A
*Proton motive force from linear electron flow only*	*pmf_LEF_*	0.484 ± 0.033 A	**0.344 ± 0.016 B**	0.482 ± 0.035 A
*Total light-dark Δ pmf (×1,000)*	*ECSt*	0.627 ± 0.055 A	**0.453 ± 0.020 B**	0.603 ± 0.066 AB
*Lifetime of steady-state ATP synthase proton efflux (×1,000)*	*ECS*	7.476 ± 0.536 A	**5.543 ± 0.220 B**	7.682 ± 0.538 A
*Oxidized PSI centers, where acceptors lack electrons*	*PSI_ox_*	0.237 ± 0.027 A	0.166 ± 0.053 AB	**0.114 ± 0.029 B**
*Over reduced PSI centers, where acceptors are saturated with electrons*	*PSI_or_*	0.276 ± 0.084 B	**0.551 ± 0.050 A**	**0.557 ± 0.033 A**
*Open PSI centers that are ready to accept electrons*	*PSI_o_*	0.669 ± 0.093 A	0.382 ± 0.067 AB	**0.313 ± 0.047 B**
*Active PSI centers that are “operational” to receive/pass electrons*	*PSI_A_*	1.390 ± 0.088 B	1.597 ± 0.074 B	**1.959 ± 0.078 A**
*Relative chlorophyll content*	*SPAD*	50.44 ± 1.969 B	54.23 ± 0.810 B	**58.48 ± 0.955 A**

Note that the dataset originally contained the constant-light treatment, but it was excluded for this analysis. The data were highly variable, and the leaves were visibly unhealthy/chlorotic. It was determined that 3 weeks of constant treatment causes damage so extensive that the MultispeQ data do not provide information on the imbalances that caused the injury (particularly electrochromic shift and photosystem 1 absorption-based methods). Means and standard error (n = 8) are presented with letters to denote if a significant difference was found using least square means with Tukey–Kramer adjustment (p< 0.05), same letters are not significantly different from each other.

All plants, which have been acclimating to their respective lighting treatments for 3 weeks, were transferred to the same growth chamber during mid-day to measure at steady state under similar conditions.Values that are significantly different are bolded (in addition to ascribed letters that denote significance) to make it easier to see.

Quantum yield of PSII (ϕ_PSII_) is significantly lower in both dynamic treatments than control. However, the fraction of dissipated energy as regulated NPQ (ϕ_NPQ_) is higher in dynamic 2 than control and dynamic 1, whereas non-regulated dissipation (ϕ_NO_) is higher in dynamic 1 than control, but not different from dynamic 2.

Absorption-based parameters give further information on thylakoid dynamics. Dynamic 1 had a significantly higher steady-state proton efflux/conductivity through ATP synthase (gH+) compared to both control and dynamic 2. Also, the total (light to dark) proton motive force across the thylakoid membrane (ECSt) was significantly lower in dynamic 1 than control and somewhat lower than dynamic 2 but not significantly. Relative proton flux (ν_H+_) was not significantly different between any treatments, although dynamic 2 seemed lower.

Combining fluorescence and absorption-based parameters gives some more relationships to explore. The proton motive force from LEF only (pmf_LEF_) was lower in dynamic 1 than both control and dynamic 2. However, proton pumping by CEF (ν_H+_ LEF^−1^) was not significantly different between any treatment. In addition, ECSt maintained the same relative relationship with the lowered pmf_LEF_, again indicating that CEF did not significantly increase. However, there is one limitation to the comparisons found through the relationship between pmf_LEF_ and ν_H+_ being different in dynamic 1 compared to both control and dynamic 2. This can either indicate a pigment composition change or differing ECS response. Considering dynamic 2 has a differing pigment composition (SPAD), it may be difficult to draw conclusions for it. It is also important to reiterate that dynamic 2 had some very mild injury, but it did provide information on imbalances compared to control and dynamic 1.

Finally, the analysis of the electron transport chain can be completed by observing the oxidation state of PSI centers using the absorption-based methods programmed into the RIDES 2.0 protocol of the MultispeQ. Both dynamic 1 and dynamic 2 had significantly higher fraction of over reduced PSI centers than control. However, dynamic 1 PSI oxidized centers and PSI open centers were not significantly different than control, whereas dynamic 2 was. Nonetheless, dynamic 1 seemed to be under slightly less pressure than dynamic 2. Dynamic 2 also had higher active PSI centers than control, and dynamic 1 was between the two showing no significant difference either way. Combined with the fact that qL was significantly lower in dynamic 1 than control (and dynamic 2 was lower but not significantly than control) and the differing gH+/NPQt responses, we can interpret that both dynamic 1 and dynamic 2 had more reduced electron transport chains than control, and they each engaged different mechanisms to deal with it.

### 3.5 Short-term diurnal MultispeQ patterns under different lighting treatments in tomato

**Figure 7:**
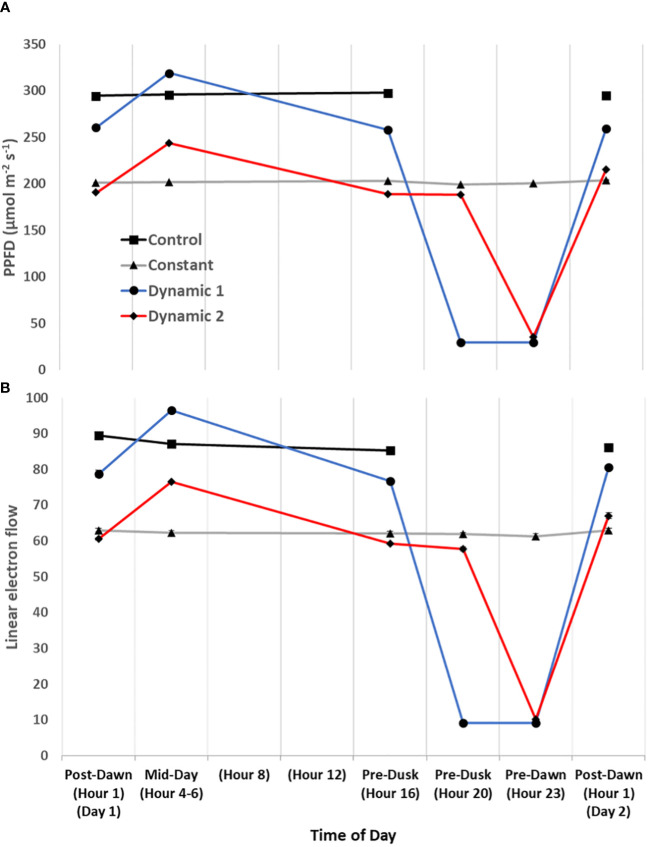
Diurnal light intensity and linear electron flow. The diurnal time-course of tomato measured under their respective treatment conditions for the first day. Actinic light intensity used in fluorescence- and absorption-based protocols is plotted as ambient light intensity **(A)**. Overall, linear electron flow (LEF) **(B)** responds as expected to light intensity, but there does seem to be a subtle decrease of LEF over time of day that is most noticeable in control. Each measurement was an average of three technical replicates for each biological replicate in a repeated measures design. Mean and standard error from n = 4.

**Figure 8:**
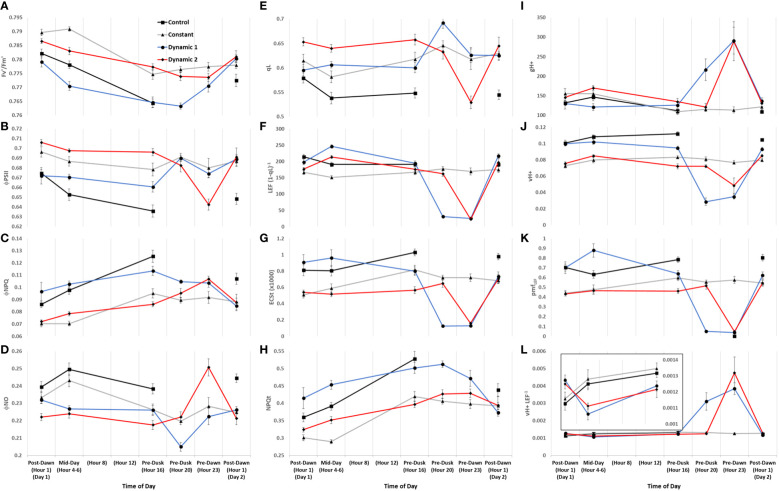
Diurnal MultispeQ fluorescence- and absorption-based parameters during the first day of tomato plants exposed to light treatments. Estimated parameters across all light treatments show strong diurnal patterns with notable treatment effects. PSII maximum efficiency (Fv’/Fm’) **(A)**, PSII operating efficiency (Φ_PSII_) **(B)**, quantum yield of non-photochemical quenching (Φ_NPQ_) **(C)**, quantum yield of non-regulated dissipation (Φ_NO_) **(D)**, fraction of PSII open reaction centers (qL) **(E)**, apparent conductance of cytochrome b_6_f to linear electron flow [LEF (1 − qL)^−1^] **(F)** (Johnson et al., 2021), total light-dark proton motive force (ECSt) **(G)**, light adapted non-photochemical quenching NPQt **(H)** (Tietz et al., 2017), ATP synthase activity (gH+) **(I)**, relative proton flux (ν_H+_) **(J)**, proton motive force driven by linear electron flow (pmf_LEF_) **(K)**, and cyclic electron flow (ν_H+_ LEF^−1^) **(L)**. Overall, we can summarize that upstream NPQt regulatory processes act distinctly from qL/Φ_NO_-related quenching processes, the former being dependent on the duration of the photoperiod and light intensity shifts, whereas the latter showing an interesting circadian gating phenomenon. We can also highlight that nighttime under dim-light promotes high levels of cyclic electron flow regardless of feedback inhibitions reflected in qL or degree of NPQt. Plants were analyzed using a repeated measures design showing mean and standard error from n = 4.

Upon the first day of tomato plants being transferred to their respective treatments (from a shared growth chamber), a time-course series of MultispeQ measurements were taken under *in situ* conditions (**Figures 7**, **8**). “Post-Dawn Hour 1 Day 1” was taken 1 h to 2 h after transfer/start of the photoperiod. “Mid-Day Hours 4–6” was taken between 4 h and 6 h into the photoperiod, with the range implying that dynamic 1 and dynamic 2 were measured near the end of their respective high blue light phase to capture the full effect of duration (control and constant were measured between them). “Hour 8” and “Hour 12” did not have any measurements, rather they are shown to ensure the x-axis time points are evenly spaced. “Pre-Dusk Hour 16” was measured just prior to the end of the control photoperiod (<16 h), whereas “Pre-Dusk Hour 20” represents the end of the photoperiod for dynamic 2 treatment (<20 h). “Pre-Dawn Hour 23” was measured prior to the start of the next photoperiod (<24 h). “Post-Dawn Hour 1 Day 2” was measured 1 h to 2 h after the start of the next photoperiod, 24 h after post-dawn (day 1).

Generally, for all treatments, PSII maximum efficiency (Fv’/Fm’) (A) tends to decrease to its lowest values by the end of the acclimated photoperiod (Pre-Dusk Hour 16) with absolute values largely dependent on light intensity. The impact is seen in a likewise decline of PSII operating efficiency (Φ_PSII_) (B) over the photoperiod, largely explained by increases in regulated dissipation of excitons through NPQ (Φ_NPQ_) (C) and NPQt (H). However, non-regulated dissipation (Φ_NO_) adds an independent source of diurnal variation to Φ_PSII_ through changes in the fraction of PSII open reaction centers (qL), which is indicative of basal/dark quenching. At mid-day, under unchanging light of control and constant, there is an increase in Φ_NO_ (decrease in qL) that is mitigated by acclimation to an increase of blue light intensity in both dynamic treatments (this is not intuitive as qL generally decreases with increases in light intensity). As the photoperiod is extended beyond Hour 16 for constant, it seems there is a slight rhythm in Φ_PSII_ and Φ_NO_/qL, whereas Φ_NPQ_, but more so NPQt, plateaus at Pre-Dusk Hour 16, only slightly declining over the rest of the night into the next day. For both dynamic treatments, Fv’/Fm’ (with NPQt) does not recover for the first 4 h of the low-light portion of their respective nighttime spectral treatments and, in both cases, recovers most prominently upon re-introduction to “day spectrum” from Pre-Dawn Hour 23 to Post-Dawn Day 2 Hour 1. The total light-dark proton motive force (ECSt) (G) agrees well with NPQt for control and constant during the acclimated photoperiod, indicating that luminal pH was driving NPQt as expected (although we cannot officially differentiate quenching components with this protocol). However, during Pre-Dusk Hour 20 and Pre-Dawn Hour 23 and just Pre-Dawn Hour 23, when dynamic 1 and dynamic 2 were in their subjective nights, respectively, ECSt was no longer associated with NPQt. NPQt remained high, whereas ECSt dropped, showing a persistent form of photoinhibition rather than a quick reversible quenching. ECSt must have dramatically dropped thanks to a large drop in light intensity that decreased relative proton flux (ν_H+_) (J) along with an increase in ATP synthase activity (gH+) (I). There was also a profound three- to five-fold increase in proton pumping mediated by CEF (ν_H+_ LEF^−1^) (L) that aligns closely with the increased gH+. A drop in proton motive force driven by LEF (pmf_LEF_) (K) and a five-fold drop in apparent conductance of cytochrome b_6_f to LEF [LEF (1 − qL)^−1^] (F) confirm a transition from LEF to CEF once low-light nighttime treatment started regardless of timing. Overlaid on this light-dependent recovery mechanism was a time-dependent mechanism. Dynamic 1 had an advantage, much more than dynamic 2, between Pre-Dusk (Hour 16) and Pre-Dusk (Hour 20) that shows there was a conditional and different kind of recovery through Φ_NO_/qL when transferred into low light. Dynamic 2 seems to have missed this Φ_NO_/qL window and instead displays the complete opposite response when it is transferred into low light. Both dynamic LED responses, considered together but in shifted phases, indicate a nighttime recovery from a non-regulated quenching mechanism (and a susceptibility to it) that seems to be gated, creating a coincidence between circadian rhythm and metabolism fluctuation. The background circadian signal can be seen in the slight Φ_NO_/qL rhythm in constant at the same phases and the dramatic increase of Φ_NO_/qL in dynamic 1 at the Pre-Dusk Hour 20 phase. This is a significant finding, as the same light treatment effect (shifting to low nighttime light intensity) would be expected to give a similar metabolic response regardless of phase, but, here, we see that they amplify a background circadian rhythm instead. Aside from these large happenings at subjective night, during mid-day, both dynamic LED treatments drop ν_H+_ LEF^−1^ during their blue light additions, again likely responding to light intensity. However, the dynamic treatments differ from each other in pmf_LEF_, with dynamic 1 having a large increase. There was also a small increase in gH+ and ν_H+_ in dynamic 2 but not in dynamic 1. It could be that dynamic 2 was suffering from proton leakage (Avenson et al., 2005). This can be interpreted as optimal duration of high blue light that has an observable beneficial effect at<3 h but could be detrimental after<5 h. Interestingly, ν_H+_ LEF^−1^ appears to increase toward the end of the photoperiod in constant and control but reaches a maximum pre-dusk (Hour 16) and slightly drops as the photoperiod extends.

One of the most notable results that is relevant to photorespiration and daily ATP budgeting is the large relative increase of cyclic electron transport during low light at subjective nighttime for both dynamic treatments.

### 3.6 Short-term diurnal MultispeQ patterns under constant light comparing tomato and mini-cucumber

**Figure 9:**
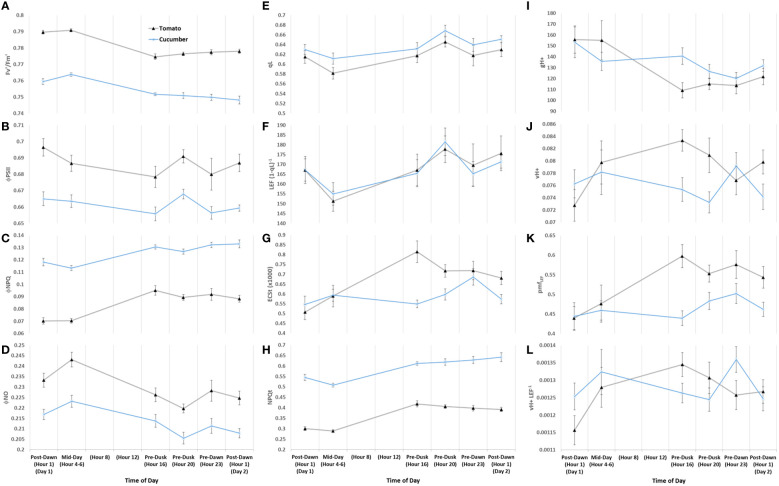
Diurnal combined absorption- and fluorescence-based parameters of tomato and mini-cucumber under the first day of constant light. PSII maximum efficiency (Fv’/Fm’) **(A)**, PSII operating efficiency (Φ_PSII_) **(B)**, quantum yield of non-photochemical quenching (Φ_NPQ_) **(C)**, quantum yield of non-regulated dissipation (Φ_NO_) **(D)**, fraction of PSII open reaction centers (qL) **(E)**, apparent conductance of cytochrome b_6_f to linear electron flow [LEF (1 − qL)^−1^] **(F)** (Johnson et al., 2021), total light-dark proton motive force (ECSt) **(G)**, light adapted non-photochemical quenching NPQt **(H)** (Tietz et al., 2017), ATP synthase activity (gH+) **(I)**, relative proton flux (ν_H+_) **(J)**, proton motive force driven by linear electron flow (pmf_LEF_) **(K)**, and cyclic electron flow (ν_H+_ LEF^−1^) **(L)**. These patterns are indicative of an endogenous circadian rhythm of non-regulated quenching, which is remarkably similar in both unrelated species. Plants were analyzed using a repeated measures design showing mean and standard error from n = 4.

Initial fluorescence-based parameters show nearly identical response patterns during the first constant day between tomato ‘Money Maker’ and mini-cucumber ‘Beesan’ (**Figure 9**). The most remarkable difference between tomato and cucumber can be seen in ECSt patterns (G). Firstly, ECSt, which can be associated with luminal pH, is closely linked with NPQt (H) in tomato, whereas it is not associated in cucumber. This shows that tomato is engaging a fast-relaxing quenching responses over the acclimated photoperiod, whereas cucumber is accumulating slow-relaxing photoinhibition. In fact, cucumber has a constitutively higher Φ_NPQ_ (C)/NPQt to begin with, showing that this species has an inherently higher photoinhibition that tomato in our system. The separation of NPQt response from ECSt may be attributed to cucumber’s ability in maintaining ATP synthase activity (gH+) (I) longer than tomato. It is not until the photoperiod is extended past its acclimated amount (Pre-Dusk Hour 20) when gH+ begins to drop causing ECSt along with NPQt to rise in cucumber. Interestingly, CEF (ν_H+_ LEF^−1^) (L) has a peak at Pre-Dawn Hour 23 in cucumber, whereas tomato seems to have upregulated CEF earlier and then downregulates it at that time. Cucumber uniquely seems to build up pmf Pre-Dawn through increases in CEF and other LEF mechanisms without a matched ATP synthase activity for proton efflux until the subsequent Post-Dawn when the issue resolves. In both species, there is an ephemeral increase in Φ_NO_ (D) at Mid-Day Hours 4–6, a dip at Pre-Dusk Hour 20, and a return to base-level at Pre-Dawn Hour 23. The opposite pattern is reflected in open PSII reaction centers (qL) (E) and cytochrome b_6_f conductance to LEF [LEF (1 − qL)^−1^] (F). These last patterns are indicative of an endogenous circadian rhythm of non-regulated quenching, which is remarkably similar in both unrelated species.

## 4 Discussion

### 4.1 Using dynamic LEDs to guide canopy architecture and biomass partitioning

Dynamic LEDs, which can change spectra and timing, offer a flexible system that can be tailored to the plant growth objectives needed. Our objectives were to extend the photoperiod without compromising canopy architecture and inducing photoperiodic injury. Dynamic LEDs enabled a successful photoperiod extension strategy by starting with a base circadian entrainment program that includes a timed “peak spectrum” overlayed on a “day spectrum” and then a transition into a “night spectrum.” The strategy allows for flexibility in dosing the “peak spectrum” and “night spectrum” cues independently to adjust canopy architecture.

The “peak spectrum” consisted of a short duration (3 h to 5 h) high blue light enrichment during late morning/afternoon. The discrete signal was intended to mimic the natural increase in high light/blue light of the solar spectrum at mid-day, when the circadian rhythm would have an anticipated sensitivity to it. Rather than a homogenous increase in light intensity from the LED fixture, which is costly, we attempted to mimic a strong high light response by focusing all the energy into blue light. Blue light is known to induce the signal for short- and long-term acclimation responses (Hogewoning et al., 2010; Hoffmann et al., 2015; Huché-Thélier et al., 2016: Kang et al., 2021). Blue light also stimulates stomatal opening, which is important for balancing solar radiation energy input with transpiration driven energy output (Geelen et al., 2019; Marie et al., [In press]). Also, blue LEDs are efficacious, contributing to a higher total LED fixture efficacy if the fixture has a relatively higher proportion of blue LEDs than white LEDs (Kusuma et al., 2020).

However, there are drawbacks to how much blue light should be added in a growth spectrum, as excessive blue light from artificial lighting sources can cause photoinhibition and leaf damage, likely from the combination of photosensitizers in the electron transport chain/chlorophyll that produce damaging singlet oxygen and the over-excitation of PSII water-splitting manganese complex that releases manganese ions in the lumen acting as inhibitors in other PSII reaction centers (Zavafer and Mancilla, 2021). However, more likely at the levels that we are proposing, too much relative (and absolute) blue light can create an overly compact plant architecture that reduces canopy radiation capture (Snowden et al., 2016; Kaiser et al., 2019; Kalaitzoglou et al., 2021). Extending the photoperiod also aggravates the compactness problem (Warner et al., 2023). Therefore, compensating for these two impacts on canopy architecture is a basic requirement for a successful dynamic LED strategy to be integrated with practical management practices.

Far-red light has the opposite effect by inducing stem elongation and leaf expansion to varying degrees in most species, collectively termed the shade-avoidance response (Demotes-Mainard et al., 2016). Adding far-red to a blue-rich spectrum during the photoperiod also results in an interesting interplay of counteracting morphological and photosynthetic responses (Meng and Runkle, 2019; Kong and Nemali, 2021). If far-red is applied during the photoperiod, then the effects on canopy morphology are dependent on total light intensity, but it is not a general rule across all species (Kusuma and Bugbee, 2023). Far-red can also induce morphological effects whether supplied during the photoperiod or at end of day (EOD) (Kalaitzoglou et al., 2019). Concentrating the full dose of far-red at EOD drives a stronger response than if spread throughout the photoperiod, and the effect is even stronger if given after the photoperiod (Zou et al., 2021).

Far-red supplied during the photoperiod, from a photosynthetic point of view, not only is beneficial when combined with other spectra for driving assimilation (Zhen and Bugbee, 2020) but also has photoprotective effects under fluctuating high light conditions (Kono et al., 2017). However, far-red applied throughout the photoperiod decreases expression of morning circadian genes and increases expression of evening genes, resulting in suppressed amplitude of transcript rhythms (Wenden et al., 2011). In addition, while far-red induces useful generative behavior in greenhouse tomato, it also increases susceptibility to disease if supplied throughout the photoperiod (Ji et al., 2019; Kim et al., 2019; Meijer et al., 2023).

Therefore, there is an upper limit on how much far-red can be added in a photoperiod, necessitating the reliance on EOD far-red to counteract most of the blue light plus extended photoperiod induced morphological responses. An example of this was found in greenhouse pepper, which becomes overly compact under continuous light but was completely alleviated if far-red was added to the nighttime phase of the alternating LED spectrum (Lanoue et al., 2022). These considerations informed the implementation of far-red in addition to dim-blue during the nighttime spectra.

The effects from dynamic LED treatments were uncertain because there is not an extensive database for greenhouse crops under dynamic changing spectra. Differences in biomass partitioning, particularly, were under question as the far-red induced shade avoidance response was needed for plant height gains but is commonly at the expense of leaf mass per area (LMA) (Casal, 2012; Chitwood et al., 2015). Additionally, dim-blue light at nighttime engages an additional shade-avoidance response through phototropins (Kong and Zheng, 2020). Interestingly, the short 3-h pulse of blue light at early to mid-day in dynamic 1 treatment on tomato was sufficient to counteract the leaf-level LMA shade-avoidance response, all while not impacting the stem-level aspect of the response in tomato (**Figure 2**; **Table 1**). The segregation of LMA and leaf area from plant height in dynamic 1 proved to be valuable for total biomass gains and an ideal canopy architecture. Mini-cucumber under dynamic 1, however, did not differ from control morphologically except with an increased partitioning to petioles, which may have improved canopy architecture.

Dynamic 2 in tomato had a significantly higher LMA and no difference in plant height compared to control. The increased partitioning of dry weight to leaves, increased LMA, decreased leaf area, decreased plant height, and decreased partitioning to stem in dynamic 2 clearly follows the trend of increased daytime blue light fraction found in another study using similar aged tomato transplants and treatment duration (Kalaitzoglou et al., 2021). Compared to dynamic 1, the increase in LMA was most likely associated with the longer 5-h pulse of blue light. However, dynamic 1 and dynamic 2 had equal blue and far-red DLI doses, demonstrating that timing played a major role in the differing responses. Mini-cucumber seemed much more sensitive to the blue light timing, showing decreases in plant height and leaf area along with an increase in LMA. Clearly biomass partitioning was diverted away from stem fraction and put into leaf fraction, but oddly also in petiole fraction. This suggests that nighttime far-red timing plays a stronger role on petiole morphology than stem morphology, whereas blue light timing mid-day has a stronger impact on stem morphology in mini-cucumber ‘Beesan.’

The optimal dynamic LED recipe for mini-cucumber still needs to be devised as the presented experimental design did not thoroughly explore all timing and dosing options. In addition, these differing responses across greenhouse crops highlight the importance of the need for flexibility in supplemental lighting strategies. In this work, we presented a small case study where the same dynamic LED formula induced profound canopy differences by tuning minor blue and far-red timing variations. These variations can certainly be optimized on a crop-by-crop basis (and even adjusted on a weekly basis as needed by the grower in tandem with existing dynamic temperature control strategies).

### 4.2 Dynamic 1 exhibits potential for increased yield in mini-cucumber

Unexpectedly, mini-cucumber yield (from unpruned plants) was significantly higher in dynamic 1 (93.77 g ± 13.35 g) than control (70.42 g ± 13.36 g), and far greater than constant (31.17 g ± 13.36 g) (**Table 2**). However, total biomass, leaf area, and height were not significantly different than control (**Table 2**), which is a stark contrast to the responses seen in tomato. Although, there was a subtle morphological difference that is discernable in visual appearance of the plants (**Figure 2**), which may be partially explained by a greater biomass partitioning to petioles in dynamic 1 (**Table 2**). Interestingly, not only yield was greater in dynamic 1, but it also had a much greener fruit, contributing to a higher shelf appeal in terms of fruit quality (dynamic 2 shared this response) (**Figure 3**). These results were not expected as the similar red/dim-blue alternating LED strategy in a greenhouse experiment demonstrated no net-positive effects on mini-cucumber yield compared to constant or control (Lanoue et al., 2021). Our differing results are most likely due to the differences between greenhouses and growth chambers. However, it would be worth trying nighttime far-red in the greenhouse (as an optimization of the existing alternating red/dim-blue strategy), as that was never done before and shows promise for increasing mini-cucumber yield from our growth chamber study. Also, the addition of short-duration mid-day blue in greenhouse production may be beneficial for enhancing fruit quality (greenness), especially in winter when several consecutive cloudy days limit blue light from natural sunlight.

### 4.3 Successful acclimation to extended photoperiods depends on sustained photorespiration

At present, there are no explanatory stress markers for photoperiodic injury other than reductions in Fv/Fm, which represents a general photoinhibition. First, we assessed fast screening methodologies to see if we could define possible mechanisms easily. Fortunately, after 3 weeks of treatment, we were able to see a very mild injury developing in dynamic 2, which served as a much better comparison to healthy control and dynamic 1 than the excessively injured constant treatment. Indeed, the constant-light treatment had a much lower Fv/Fm, which we interpret as the late stages of photoinhibition, but dynamic 2 did not exhibit any measurable photoinhibition.

A quick comparison between net assimilation rates, respiration in the dark, Fv/Fm, and quantum yield of PSII (YII) under ambient growth conditions shows no significant differences between control, dynamic 1, and dynamic 2 for tomato (**Figures 4**, **5**). Also, identical treatment comparisons were made for mini-cucumber ‘Beesan’ (**Figures 4**, **5**) and a photoperiodic injury–tolerant tomato genotype ‘UofGPIT’ grown under constant (**Supplementary Materials 3–6**). To dig deeper, we implemented a high-throughput screening method for photorespiration rate (V_O_/V_C_) (Bellasio et al., 2014) under ambient conditions (air temperature 21°C, PPFD 300 µmol m^−2^ s^−1^, C_a_ 440 µmol mol^−1^). Increases in V_O_/V_C_ were hypothesized to ameliorate stress induced by extended photoperiods in both dynamic treatments. For photoperiodic injury–sensitive tomato ‘Money Maker,’ we seen a significant increase of V_O_/V_C_ in dynamic 1 (0.257 ± 0.016) compared to control (0.207 ± 0.007). Dynamic 2, on the other hand, did not have a significant increase of V_O_/V_C_ (0.237 ± 0.007) compared to control (although its value was in between control and dynamic 1) (**Figure 6**). The original intention of dynamic 2 was to improve the electrical cost efficiency of the alternating 12-h/12-h red/dim-blue introduced by Lanoue et al. (2019) by extending the “daytime” photoperiod to 20-h/4-h. However, the presented configuration of dynamic 2 pushed the limits, and we can use this opportunity to find out why.

Interestingly, V_O_/V_C_ was significantly greater than control in dynamic 1, dynamic 2, and constant for the photoperiodic injury–tolerant species mini-cucumber ‘Beesan.’ Furthermore, a photoperiodic injury–tolerant tomato genotype ‘UofGPIT’ grown under constant light also displayed a higher V_O_/V_C_ (**Supplementary Material 6**). Unexpectedly, the photoperiodic injury–tolerant tomato cultivar ‘UofGPIT’ and mini-cucumber ‘Beesan’ had nearly the same photorespiration level under constant light (0.295 ± 0.003 and 0.284 ± 0.014, respectively). Also, under the dynamic 1 LED treatment, photorespiration was nearly the same between photoperiodic injury–sensitive tomato ‘Money Maker’ (0.257 ± 0.016) and tolerant mini-cucumber ‘Beesan’ (0.256 ± 0.014). The comparisons may be justified by the fact that control had similar levels between tomato ‘Money Maker’ (0.207 ± 0.007) and mini-cucumber ‘Beesan’ (0.196 ± 0.014). These results are highly suggestive that photoperiodic injury tolerance derived from both adaptation (across unrelated species/tolerant genotypes within species) and acclimation (using dynamic LEDs) involves the upregulation of photorespiration.

The fact that tomato ‘Money Maker’ was displaying a very mild form of photoperiodic injury under dynamic 2 and was found to not upregulate photorespiration to the degree that mini-cucumber did under dynamic 2 (unlike their similarity under dynamic 1) can point toward a downstream limitation. The MultispeQ was used to further explore this limitation in 3-week acclimated tomato (**Table 3**). The light-dark difference in total proton motive force (ECSt) (also related to luminal pH) and conductance of protons through ATP synthase (gH+) together indicate dynamic 1 had either a more sensitive ATP synthase activity (possibly a higher P_i_ substrate availability) and/or more abundant ATP synthase content in the thylakoids than control and dynamic 2 (Avenson et al., 2005). The notion of a higher ATP synthase content/activity in dynamic 1 is supported by the lack of additional NPQt above control, meaning the proton efflux through ATP activity/content was able to maintain luminal pH within a healthy non-dissipative inducing range that was useful for ATP: NADPH balancing (type I response) (Kramer and Evans, 2011). Whereas dynamic 2 did not maintain a healthy ATP synthase activity/content that did not enable appropriate proton efflux, observed as a lower gH+ and higher ECSt, which caused a significant induction of NPQt (type II response) (Kramer and Evans, 2011).

Dynamic 1 also had a lower fraction of proton motive force from LEF (pmf_LEF_), but it was not due to an increase in CEF (ν_H+_ LEF^−1^), rather it was due to the ease of proton efflux through ATP synthase, which did not need as much pmf (Takizawa et al., 2008). Therefore, the difference in ATP synthase activity could be due to P_i_ substrate availability, being limiting in dynamic 2 but not limited in dynamic 1, causing the buildup of protons in dynamic 2. This is supported by the finding that photorespiratory P_i_ substrate–alleviating qualities are deficient in dynamic 2, implying a cause and effect.

The upstream question remains, for tomato ‘Money Maker’ dynamic 2, what caused a failure to fully upregulate photorespiration yet maintain a high carboxylation capacity? Many photorespiratory genes/enzymes are regulated by light and metabolic feedback signals (Aroca et al., 2023). One interesting negative feedback regulator of photorespiration is an increase in serine pools, which has been shown to selectively inhibit transcription of photorespiratory genes (Timm et al., 2013). In addition, glycine decarboxylase in the mitochondria, responsible for the conversion of glycine to serine, is regarded as the central modulator of photorespiratory flux, which can exert immediate control via post-translational modifications (Timm and Hagemann, 2020). The serine-to-glycine ratio downregulates photorespiration if high and upregulates it if low (Timm et al., 2016). Serine has been described to interconnect S, N, and C1 metabolism and be involved with stress acclimation (Aroca et al., 2023). In addition, although photorespiration accounts for most of the serine production in plants, two other glycolysis-branch serine pathways are engaged during stress, act in non-photosynthetic tissues, and are allosterically inhibited by serine, and many mutations in these pathways are embryo lethal that implicates glycine to serine ratio as having a crucial role in primary metabolism (Igamberdiev and Kleczkowski, 2018). Future research could measure photoperiod dependent accumulation and export of glycine/serine pools that are possibly associated with selective suppression of photorespiration (i.e., without affecting RuBP carboxylation) and the hypothesized differences in export over the nighttime spectra of dynamic LED recipes (along with simple photoperiod extension).

Importantly, selectively inhibiting photorespiration does not relax associated ATP-compensating mechanisms that were originally engaged with it (Smith et al., 2023). For example, exposure to low O_2_ increased lumen acidification, which was attributed to a decrease in apparent ATP synthase activity caused by an ATP surplus (i.e., suddenly reducing photorespiration will drop ATP consumption and lead to another form of TPU/P_i_ limitation) (Smith et al., 2023). Regarding dynamic 2, it could be that excessive serine was suppressing photorespiration, which caused a build up of unused ATP that subsequently led to a P_i_ limitation/ATP synthase activity bottleneck.

### 4.4 Short-term acclimation mechanisms under dynamic LEDs

TPU limitation was reported to occur upon the first day of photoperiod extension in rice grown in a controlled environment (Fabre et al., 2019). Once TPU is reached, there is an immediate imbalance in P_i_ availability, causing dynamic changes in redox states (McClain et al., 2023). We observed that early stages of acclimation to extended photoperiod (and dynamic treatments) involve a time-of-day regulated redox and P_i_ balancing act, with CEF playing a huge role in driving ATP synthase during the nighttime low-light (and far-red rich) phases of dynamic LED treatments (**Figures 7**, **8**). The relative increase in ATP supply at nighttime in both dynamic treatments could be satisfying (or almost satisfying, respectively) a total daily ATP budget. Tied to ATP/proton management is the differing degree of relaxation of NPQt responses across treatments. Constant light was constitutively unrelaxed, dynamic 2 had approximately 50% recovery, whereas dynamic 1 fully recovered. Mini-cucumber seemed to have a more delayed onset of NPQt under constant light than tomato, likely due to the maintenance of ATP synthase activity for a longer duration (**Figure 9**).

Independent from the CEF and NPQt responses, a major difference between dynamic 1 and dynamic 2 redox balance can be observed during their nighttime phases. They have totally opposite responses of opening/closing PSII reaction centers (qL) due to basal/dark quenching regulation (ϕ_NO_). ϕ_NO_ represents excitation dissipation through thermal and fluorescence emission independent of NPQ, likely from closed PSII reaction centers quenching/dissipating the energy (Kramer et al., 2004; Klughammer and Schreiber, 2008). Constant-light treatment also displayed a subtle phase response, qL opening and subsequent closing 4 h later, which may point toward a circadian regulation mechanism. Interestingly, tomato and mini-cucumber share a nearly identical circadian pattern of qL and ϕ_NO_ under constant-light treatment too, showing this circadian phenomena may be conserved across unrelated species. It just so happens that dynamic 1 shifts to low light when reaction centers are opening (between Post-Dusk Hour 16 and Hour 20), emphasizing a potential peak circadian phase. Dynamic 2 shifts to low light while the reaction centers are closing (between Post-Dusk Hour 20 and Pre-dawn Hour 23), emphasizing a potential trough circadian phase. If it were purely an electron transport chain over-reduced signal, then we would expect the same qL response but differing amplitude, between dynamic LED treatments, which was not the case. This circadian gating effect inspires future experiments that could explore the link between P_i_ regulation of ATP synthase activity and CEF with the potential circadian phasing of basal/dark feedback inhibition and opening/closing of PSII reaction centers.

Short-term TPU limitation could be alleviated by an initial increase of photorespiration (McClain et al., 2023). However, TPU limitation quickly disappears after 30 h of acclimation and is balanced by downregulation of other processes (McClain et al., 2023). For example, RuBisCO is deactivated and q_E_ is engaged until long-term acclimation strategies take over (McClain et al., 2023). In a preliminary experiment, after 4 nights of continuous light, the photoperiodic injury–sensitive tomato cultivar ‘Basket Vee’ maintained higher photorespiration than control (data not shown), confirming the early onset of photorespiration and that it persists for several days, and up to/longer than 3-weeks if it can be sustained as was shown for photoperiodic injury–tolerant mini-cucumber ‘Beesan’ and tomato ‘UofGPIT.’

### 4.5 Photorespiration, peroxisomal catalase, and the circadian external coincidence model as a hypothesis for photoperiodic injury

The physiological causes and effects during photoperiodic injury are an on-going area of research. Velez-Ramirez et al. (2017b) reasoned that an ATP: NADPH imbalance resulted from the accumulation of carbohydrates and the associated decrease in Calvin cycle enzyme transcription. They found a strong correlation between carbohydrate accumulation and decreases in Fv/Fm. This supports it as a driver that induces early senescence, possibly through reactive oxygen species (ROS) derived from an over-reduced electron transport chain (Velez-Ramirez et al., 2011; 2017b). However, although carbohydrate accumulation has received a lot of attention as a cause of photoperiodic injury, it is not the full story, as other photoperiodic injury studies have not found correlations between carbohydrates accumulation and photoperiodic injury (Pham and Chun, 2020; Shibaeva et al., 2023).

We suggest it is not necessarily the accumulation of carbohydrates that causes the damage directly, rather it is initiated by TPU limitation effect on P_i_ availability. Then, the need for photorespiratory-related freeing of P_i_ substrate, as well as the consequences of photorespiration, becomes an important piece to the photoperiodic injury puzzle. The many roles photorespiration plays in balancing metabolic flux between mitochondria, peroxisome, and chloroplast are complex and offer many modes of action to investigate. However, peroxisomal H_2_O_2_ production, a by-product from glycolate oxidase’s reaction with glycolate producing glyoxylate, may be a prime candidate for ROS signaling. Furthermore, photoinhibition was found to not be directly related to photoperiodic injury (Dorais et al., 1995; Velez-Ramirez et al., 2017a). We observed over-reduced electron transport chains in both dynamic LED treatments after 3 weeks of acclimation, but dynamic 1 had no signs of injury, whereas dynamic 2 did, leading us to speculate photorespiratory H_2_O_2_ as having a more direct role.

In Arabidopsis, a photorespiration-derived H_2_O_2_ redox signal was found to be governed by a peroxisome localized *CATALASE2* (*CAT2*) in a photoperiod dependent manner, independent of light intensity and oxidative stress duration (Queval et al., 2007; 2012; Yang et al., 2019). Short-day acclimated plants show a pronounced increase in sensitivity and upregulation of oxidative marker genes in the photorespiratory *cat2* mutant (high H_2_O_2_ signal), supporting a protective glutathione antioxidant pathway and a salicylic acid–dependent antioxidant signaling pathway among others. However, long-day acclimated plants do not show this sensitivity and are unable to scavenge the excess H_2_O_2_, which then initiates programmed cell death, presumed to be a circadian rhythm mismatch (Queval et al., 2007; 2012; Yang et al., 2019). *CAT2* transcription itself is regulated by the circadian rhythm, with a peak at subjective dawn, which is dependent on the morning complex CIRCADIAN CLOCK-ASSOCIATED 1 (*CCA1*) (McClung, 1997; Lai et al., 2012). Indeed, when a *cca1* mutant was exposed to photoperiod extension stress, catalase activity was significantly lowered, and the plant became injured (Nitschke et al., 2016; Abuelsoud et al., 2020). The initiation of injury was also associated with an apoplastic increase in peroxidases, reminiscent of the oxidative burst response from pathogen infections (Nitschke et al., 2016; Abuelsoud et al., 2020). This may then have led to programmed cell death.

These studies are also relevant to tomato. Peroxisomal catalase in tomato (*SLCAT2*) expression has been shown to be upregulated during the circadian morning complex-related phase under a normal photoperiod, which shows *CAT2* in Arabidopsis and *SLCAT2* in tomato share a conserved circadian regulation pattern (Kabir and Wang, 2011). When exposed to continuous light, a tomato ‘Money Maker’ cross exhibited a constitutively lower expression of the circadian morning complex (and high expression of evening complex) (Müller et al., 2016), which could infer lower *SLCAT2* expression. For example, photorespiration and whole-leaf catalase activity were found to be higher than control when tomato plants were exposed to continuous light with the addition of temperature differentials, resulting in photoperiodic injury tolerance (Haque et al., 2017). The authors noted that there was a possible connection between peroxisomal-localized photorespiratory H_2_O_2_ release and increased catalase activity, but they were unsure of the sub-cellular localization of catalase activity. Glutathione activity was also found to be increased in this treatment, which is reminiscent of the healthy short-day response of Arabidopsis.

This leads us to hypothesize that photoperiodic injury may not be caused by absolute indiscriminate amounts of ROS, rather it could be a critical threshold of ROS during a vulnerable circadian phase. The hypothesis follows the external coincidence model of photoperiodism that has been extensively studied for flower induction (Song et al., 2015). For photoperiodic injury, the external coincidence model posits that photorespiration would be producing H_2_O_2_ in the light above a certain threshold during a circadian clock time when expression of the morning complex (with peroxisomal catalase) is low, thus initiating a programmed cell death response (akin to pathogen infection). The hypothesis is certainly testable by manipulations of the coincidence between internal circadian phase and external light signaling cue. For example, *cca1* mutant would have a constitutively lower morning complex expression and be more prone to photoperiodic injury, whereas a *toc1*-overexpressing mutant would display a similar response, both providing evidence for the circadian phase component. If a variety of photoperiodic injury–tolerant genotypes/species with these mutations showed injury, then that would be supportive evidence of its canonical nature. Non–24-h lighting (i.e., 6-h light/6-h dark and 24-h light/24-h dark) treatments have been shown to induce photoperiodic injury (Velez-Ramirez et al., 2017a), which makes sense if it follows an external coincidence model, as both treatments supply light during a sensitive phase. However, a phase-response curve of photoperiodic injury would provide definitive evidence in building the photoperiodic injury external coincidence model. We suggested that peroxisomal catalase is involved, so its activity phase response curve should be opposite to that of photoperiodic injury. Similar phase response curves of injury could be had for discrete modulations of photorespiration (elevated/lowered CO_2_) and applications of exogenous H_2_O_2_/selective catalase inhibitors.

## 5 Conclusion

Two variations of dynamic LED strategies induced differing canopy responses, opening the potential to adjust canopy architecture through counterbalances in the peak spectrum (blue) and night spectrum (far-red). Both tomato and cucumber responded well to the dynamic 1 strategy by avoiding the overly compact morphology induced by extended photoperiods. Future research will explore more variations and work on modeling the counterbalancing act for predictive programs to be applied in CEA facilities. Next, we wanted to explore a physiological foundation for successfully growing plants under continuous light. Photorespiration was hypothesized to provide a photoperiod dependent photorespiratory-P_i_ stoichiometric compensation, which would be beneficial in maintaining triose-phosphate utilization. Photoperiodic injury–tolerant mini-cucumber ‘Beesan,’ photoperiodic injury–tolerant tomato ‘UofGPIT,’ and the successful acclimation to photoperiod extension in photoperiodic injury–sensitive tomato ‘Money Maker’ (by dynamic 1 LED strategy) all displayed higher photorespiration, supporting our hypothesis. We also found that the night spectrum of dynamic LEDs promotes relatively higher engagement of CEF and ATP synthase activities that would be beneficial for the higher ATP demands of photorespiration, potentially balancing a diurnal ATP: NADPH stoichiometry. Future research could perform more in-depth modeling by using light curves and CO_2_ curves to confirm and quantify these early findings. If true, then a conceptual framework explored the possible ontology of photoperiodic injury and its relationship with photorespiration. The proposed ontology describes a photorespiratory-antioxidant balance is de-stabilized due to a circadian rhythm external coincidence model. Specifically, light-dependent photorespiratory-H_2_O_2_ is not neutralized by proper circadian regulation of peroxisomal catalase and is in a sensitive phase leading to programmed cell death/pathogen defense type response. From this multiple pathway perspective, we can explain the various types of photoperiodic injury tolerance reported in the literature. Tolerance can be achieved by proper circadian rhythm entrainment given by light cues like those found by dynamic/alternating LEDs (Lanoue et al., 2019, and the presented study), circadian entrainment by temperature cues (Ikkonen et al., 2015; Hao et al., 2017b; Haque et al., 2017), a more persistent rhythmicity of the circadian rhythm like that found in photoperiodic injury–tolerant tomato species adapted to equatorial regions (Müller et al., 2016; 2018), improved energy dissipation ability/connectivity in the LHCII like that found by restoring wildtype *CAB-13* transcription (Velez-Ramirez et al., 2014), or higher constitutive catalase activity as found in photoperiodic injury–tolerant greenhouse peppers (Murage and Masuda, 1997; Demers and Gosselin, 2002). Each species may lean more heavily on one pathway or another, but we propose the overall basal need for P_i_ substrate by pushing photorespiration is the driving factor that a particular acclimation strategy or a unique genotype adaptation must account for to deal with photoperiodic injury.

## Data availability statement

The original contributions presented in the study are included in the article/**Supplementary Material**. Further inquiries can be directed to the corresponding author.

## Author contributions

TM: Conceptualization, Data curation, Formal analysis, Investigation, Methodology, Visualization, Writing – original draft, Writing – review & editing. EL: Data curation, Methodology, Writing – review & editing. NR: Methodology, Writing – review & editing. BG: Funding acquisition, Supervision, Writing – review & editing.
