# Supplementary material for: Tomato and mini-cucumber tolerance to photoperiodic injury involves photorespiration and the engagement of nighttime cyclic electron flow from dynamic LEDs
Source: Front Plant Sci. 2024 May 22;15:1384518. doi: 10.3389/fpls.2024.1384518 (PMC11150841; doi:10.3389/fpls.2024.1384518)
Supplement: Supplementary file 1 [file DataSheet_1.docx]

Supplementary Material

**Supplementary material 1: Photosynthetic photon flux density (PPFD) and daily light integral (DLI) divided into spectral bins for each phase of light treatments.**

A central position in the biochamber at canopy height was chosen to take representative measurements using a Flame mini-spectrometer (Ocean Insight, NY, USA) calibrated for absolute irradiance, converted to photon flux, and divided into spectral bins in excel. All light treatments were set to the same total DLI (400-700nm) (17.28 mol m^-2^ d^-1^) and supplemented with the same total far-red DLI (700-800nm) (1 mol m^-2^ d^-1^). The photoperiod used to calculate DLI (or the average) is indicated below each phase column. Note, the day spectrum for control and constant has a higher intensity of blue light (resulting in higher relative percentage) (used cool-white LEDs) than the day spectrum for dynamic 1 and 2 (used warm-white LEDs) (Error! Reference source not found.C). This was done to compartmentalize blue DLI to be mostly derived in the peak phase and attempt to make blue DLI as similar as possible with control, without raising blue % in control beyond a healthy amount. The day spectra with either warm-white or cool-white also resulted in slight red and green DLI differences. The same was done for FR, however, the attempt to make them all equal was successful and within healthy ranges. Considering there are other metrics used to assess spectral quality, not all were targeted to be equal, which is an unavoidable limitation in complex dynamic LED treatment designs. Each colour bin integrated the following wavelength ranges: Blue (400-500nm), green (500-600nm), red (600-700nm), and far-red (700-800nm), PAR (400-700nm), FR750 (700-750nm), ePAR (400-750nm), FR% (*FR/FR+PAR x 100*), phytochrome photostationary state (PSS) (Sager et al., 1988), relative cycling rate (RCR) (Sager et al., 1988), the combined function *PSS - 0.2ln RCR* (PSS&RCR) (Sager et al., 1988), Blue:Red ratio, and Blue:Green ratio. Accompanying timing for each phase can be seen in Figure 1.

|  | Control | | Constant | | Dynamic 1 | | | | Dynamic 2 | | | |
| --- | --- | --- | --- | --- | --- | --- | --- | --- | --- | --- | --- | --- |
|  | "Day" |  | "Day" |  | "Day" | "Peak" | "Night" |  | "Day" | "Peak" | "Night" |  |
|  | 16hrs |  | 24hrs |  | 13hrs | 3hrs | 8hrs |  | 15hrs | 5hrs | 4hrs |  |
|  | PPFD | **DLI** | PPFD | **DLI** | PPFD | PPFD | PPFD | **DLI** | PPFD | PPFD | PPFD | **DLI** |
| Blue | 41.7 | **2.4** | 27.7 | **2.4** | 17.6 | 157.8 | 50.1 | **4.0** | 10.2 | 150.0 | 50.1 | **4.0** |
| Green | 75.4 | **4.3** | 50.7 | **4.4** | 62.6 | 63.9 | 1.0 | **3.6** | 48.9 | 49.9 | 0.9 | **3.6** |
| Red | 183.4 | **10.6** | 121.6 | **10.5** | 168.8 | 168.8 | 0.0 | **9.7** | 136.2 | 136.1 | 0.0 | **9.8** |
| Far-Red | 16.8 | **1.0** | 11.2 | **1.0** | 6.8 | 6.7 | 20.0 | **1.0** | 5.3 | 5.2 | 40.8 | **1.0** |
| PAR | 300.5 | **17.3** | 200.0 | **17.3** | 249.0 | 390.5 | 51.1 | **17.3** | 195.3 | 336.0 | 51.0 | **17.3** |
| FR750 | 13.1 | **0.75** | 8.18 | **0.71** | 4.39 | 4.53 | 17.7 | **0.76** | 3.27 | 3.33 | 36.5 | **0.76** |
| ePAR | 313.5 | **18.1** | 208.2 | **18.0** | 253.4 | 395 | 68.8 | **18.1** | 198.6 | 339.3 | 87.4 | **18.1** |
| FR% | 5.31 | **5.31** | 5.24 | **5.24** | 2.63 | 1.79 | 25.0 | **5.25** | 3.74 | 2.41 | 38.2 | **6.21** |
| PSS | 0.86 | **0.86** | 0.86 | **0.86** | 0.87 | 0.85 | 0.34 | **0.69** | 0.87 | 0.84 | 0.32 | **0.77** |
| RCR | 0.12 | **0.12** | 0.07 | **0.07** | 0.09 | 0.11 | 0.01 | **0.07** | 0.08 | 0.09 | 0.02 | **0.07** |
| PSS&RCR | 1.29 | **1.29** | 1.38 | **1.28** | 1.35 | 1.30 | 1.21 | **1.30** | 1.38 | 1.34 | 1.08 | **1.32** |
| Blue:Red | 0.20 | **0.20** | 0.25 | **0.25** | 0.12 | 0.95 | 22.4 | **0.44** | 0.12 | 1.26 | 10.5 | **0.47** |
| Blue:Green | 0.49 | **0.49** | 0.50 | **0.50** | 0.31 | 2.47 | 57.9 | **1.17** | 0.33 | 3.16 | 39.7 | **1.29** |

**Supplementary material 2: LiCor 6400 set-up and protocol used in this study.**
Prior to running protocols, fluorescence measurement parameters were calibrated (Measuring intensity = 4, Light frequency = 10kHz, filter = 5, Gain = 10) and (Multi-phase flash intensity = 10, ramp depth 40%, frequency = 20kHz, filter 50kHz). Silicone vacuum grease was applied to the gas exchange gaskets to minimize leaf mediated pores (Boesgaard et al., 2013). Block temperature was set to 21ᵒC and relative humidity was maintained at 70-80% throughout the measurement period (to approximate growing conditions). The main air intake line passed through a CO_2_ scrubber and then a humidified water bath set to 16°C dewpoint to maintain stable incoming air temperature and humidity. Specialty compressed gas cylinders containing 2% O_2_ balanced with N_2_ (NIT-OXY 2% CS K, Linde Canada Inc., ON, CAN) were also rigged in the supply air stream inlet, with appropriate splitters and overflow ball valve (overflow approximately 250cc min^-1^) ensuring both instruments received sufficient conditioned air flow. The air intake manifold included a valve that made transitioning between 2% O_2_ tank and CO_2_ scrubbed 21% O_2_ ambient air quick and easy. Both instruments were also connected to a pure CO_2_ tank (part# 9964-033, CO2 Tank Connector Block, LI-COR Biosciences, NE, USA).

The following step-by-step custom protocol was performed with the Li-Cor 6400:

At dawn, leaves were clamped and dark adapted for 20min with CO2R 440 µmol mol^-1^ and flow rate 120 µmol s^-1^, x5 measures of dark respiration were taken spaced 30sec apart.

Once dark respiration measurements were complete, set Flr Meas ON, waited until stable, then Do Fo/ Fm for dawn/ dark-adapted max quantum efficiency of PSII (Fv/Fm).

Set PAR to growth light intensity (300 µmol m^-2^ s^-1^ PPFD), 10% blue, wait for full induction and stability for 45min, take x5 survey measurement at 21% O_2_ spaced 30sec apart, Do Fs/ Fm’/ Fo’ for PSII photochemical quantum yield (YII) under ambient O_2_.

Switched air intake to 2% O_2_ cylinder, set SysUsrConsts to 2%, stabilize for 10min (ensured full flush of lines plus water bath chamber), 2% O_2_ survey measurements spaced 30sec apart, Do Fs/ Fm’/ Fo’ PSII photochemical quantum yield (YII) under low O_2_.


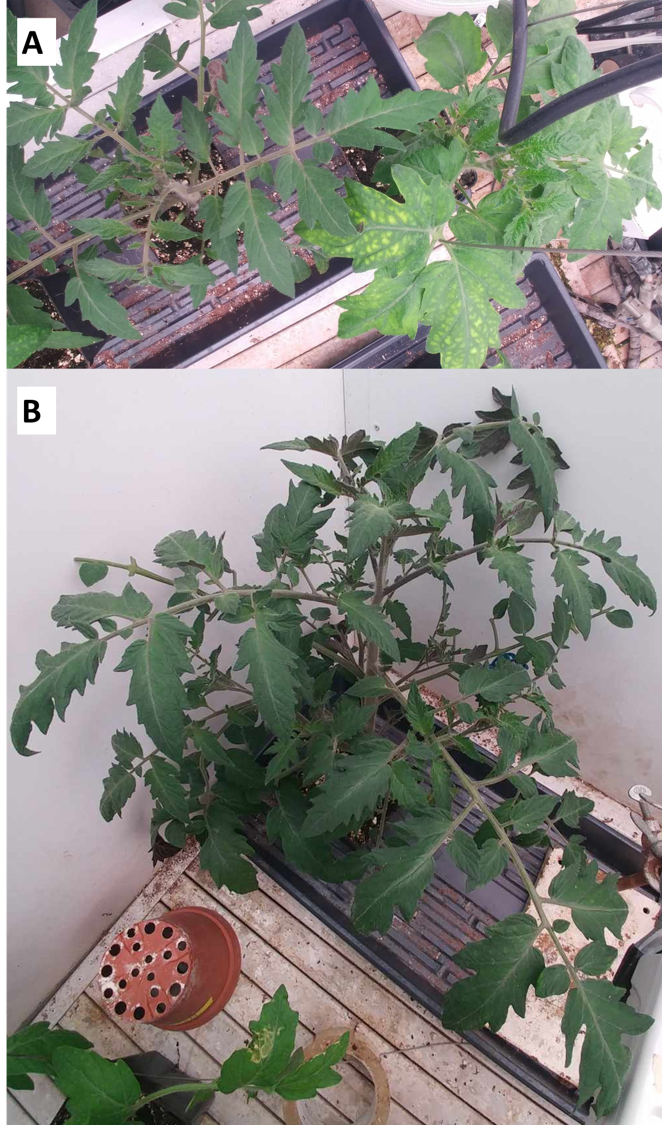


**Supplementary material 3:** A picture comparing the relative health of a photoperiodic injury tolerant tomato cultivar ‘UofGPIT’ and the intolerant cultivar ‘Money Maker’ both grown under identical constant light treatment conditions. In caption (A) ‘UofGPIT’ is on the left-hand side and ‘Money Maker’ is on the right-hand side. Both cultivars experienced 3 weeks of constant light treatment (A). After an additional couple weeks (after LiCor 6400 multicurve protocol) under constant light treatment, ‘UofGPIT’ has accumulated biomass, and its leaves continue to show no signs of injury (B). Note, ‘UofGPIT’ has a very dark green leaf and purple underside when grown under constant light, likely due to an accumulation of chlorophyll and xanthophyll pigments for increased demands on non-photochemical quenching.


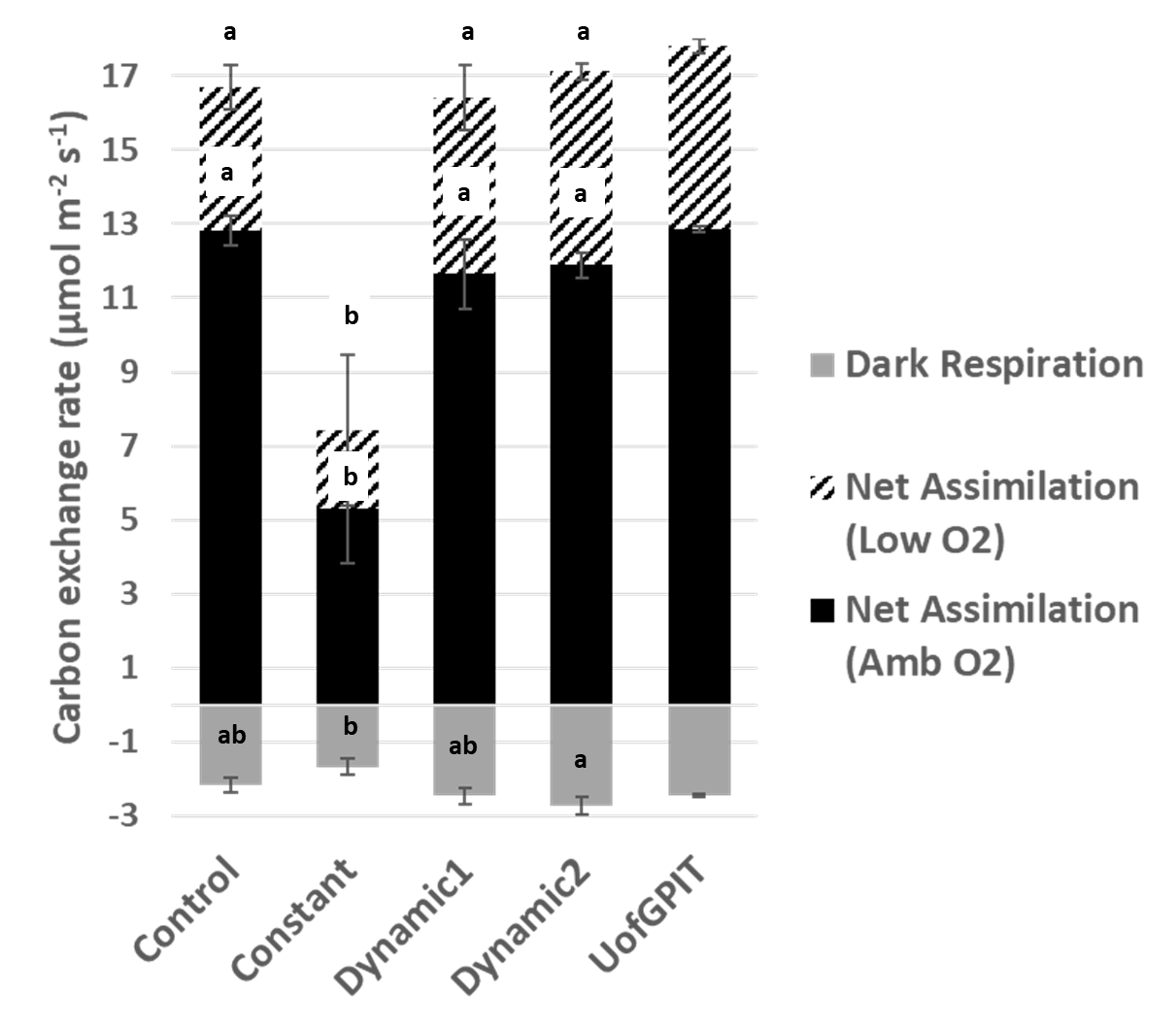


**Supplementary material 4:** Carbon exchange rates measured under ambient conditions from two tomato cultivars and one ‘Money Maker’ acclimated to different lighting treatments. The data in this figure are identical to Figure 4 tomato ‘Money Maker’ with the addition of two replicates from a photoperiodic injury tolerant tomato cultivar ‘UofGPIT’ that was grown under identical conditions as constant treatment (comparing constant light treatment on ‘Money Maker’ to constant light treatment on ‘UofGPIT’).


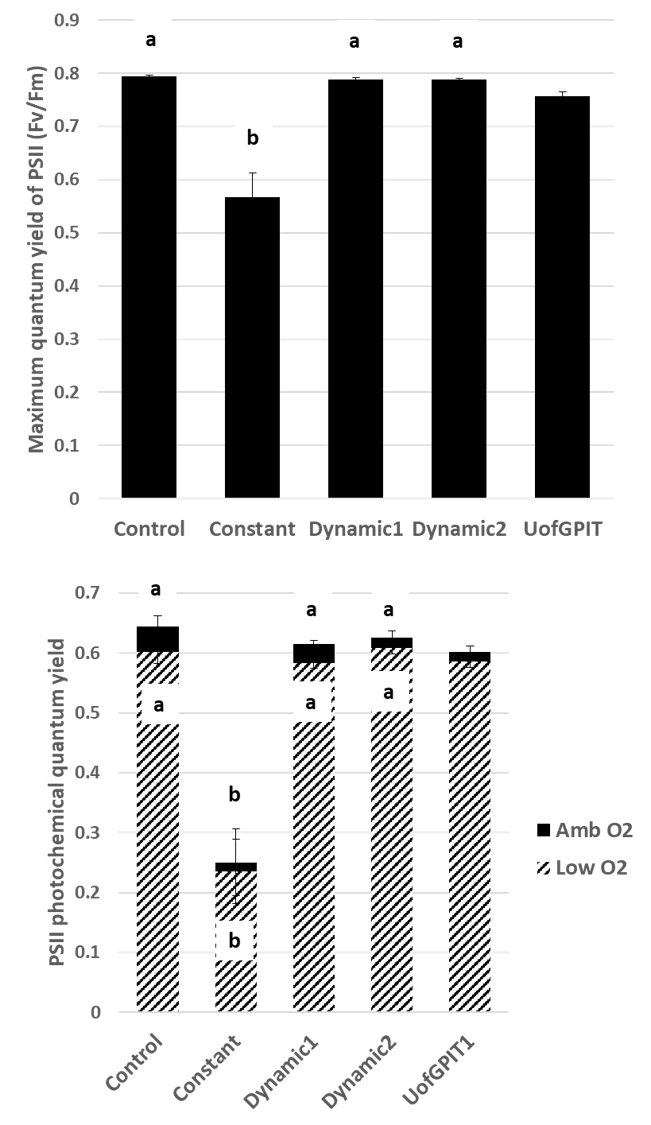


**Supplementary material 5:** Fluorescence parameters measured under ambient conditions from two tomato cultivars and one ‘Money Maker’ acclimated to different lighting treatments. The data in this figure are identical to Figure 5 with the addition of two replicates from a photoperiodic injury tolerant tomato cultivar ‘UofGPIT’ that was grown under identical conditions as constant treatment.

**
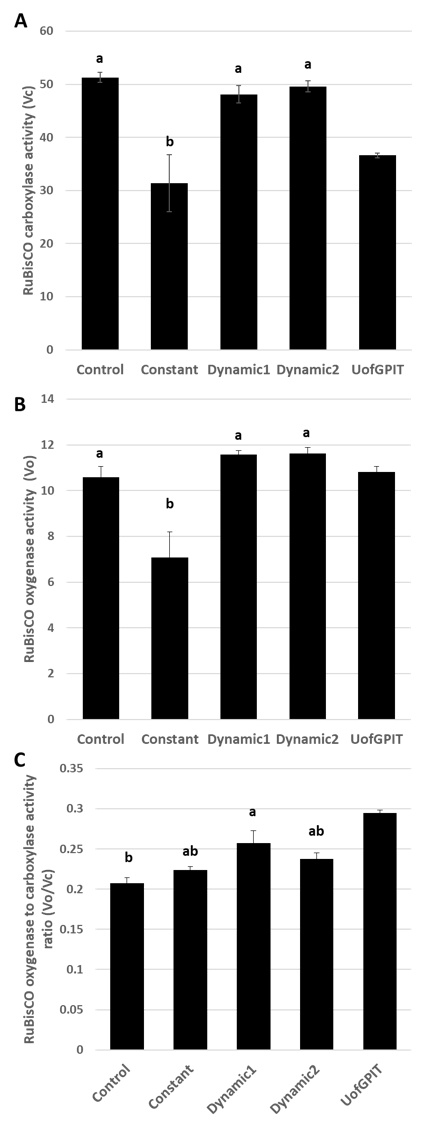
**

**Supplementary material 6:** RuBisCO carboxylase (A) and oxygenase (B) activities (V_C_ and V_O_) along with their ratio (V_O_/ V_C_) (C) under ambient conditions for two tomato cultivars and one ‘Money Maker’ acclimated to different lighting treatments. The data in this figure are identical to Figure 6 with the addition of two replicates from a photoperiodic injury tolerant tomato cultivar ‘UofGPIT’ that was grown under identical conditions as constant treatment. Interestingly, ‘UofGPIT’ has depressed V_C_ compared to control and is comparable to constant. However, ‘UofGPIT’ V_O_ is qualitatively similar to control and much higher than ‘Money Maker’ grown in the same constant light treatment. The resulting V_O_/V_C_ ratio is much higher than control and debatably higher than both dynamic LED treatments and constant. This preliminary data from a reasonably healthy photoperiodic injury tolerant tomato cultivar growing under constant light follows the hypothesis that photorespiration must be contributing to tolerance. It’s also interesting to note that mini-cucumber, a completely unrelated species, has the a similar V_O_/V_C_ ratio (~0.3) as ‘UofGPIT’ under the same constant light treatment
